# Integrating Solvent Effects into the Prediction of Kinetic Constants Using a COSMO-Based Equation of State

**DOI:** 10.1021/acs.jctc.5c00133

**Published:** 2025-03-25

**Authors:** Francisco Paes, Gabriel de Souza Batalha, Fabiola Citrangolo Destro, René Fournet, Romain Privat, Jean-Noël Jaubert, Baptiste Sirjean

**Affiliations:** CNRS, LRGP, Université de Lorraine, F-54000 Nancy, France

## Abstract

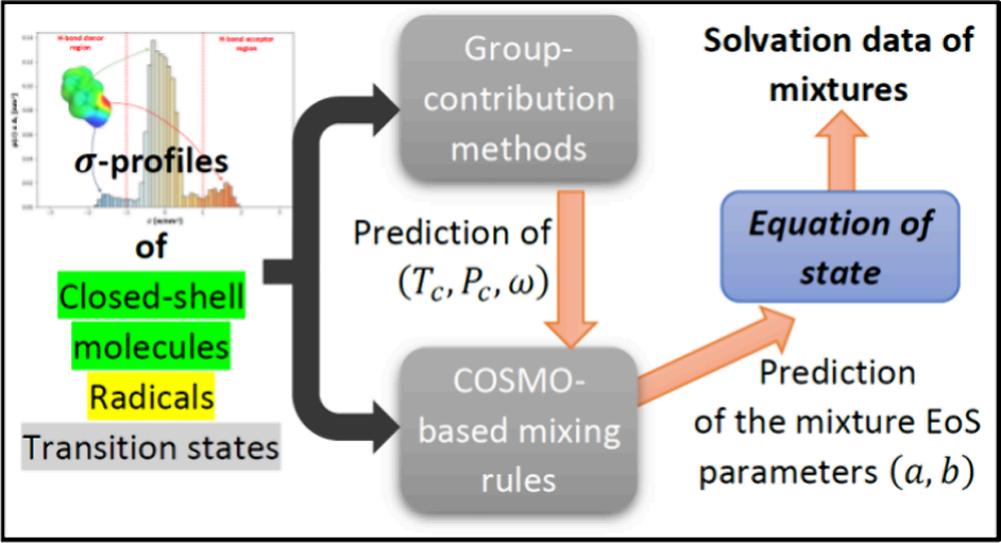

While kinetic generators produce thermo-kinetic data for detailed gas-phase kinetic models, adapting these models for liquid-phase applications poses challenges due to the need for solvent-dependent thermodynamic properties. To bridge this gap, solvation energies are used to incorporate solvent effects into gas-phase thermo-kinetic data. However, such an adaptation depends on calculating liquid-phase data of unconventional solutes such as free radicals and transition states, which are not accessible with classical equations of states. To address this issue, this work proposes a flexible framework based on an equation of state that integrates all the latest advances of this model family and is called the *tc*-PR EoS. Combined with a quantum-based continuum solvation model (COSMO-RS) through an advanced mixing rule, the proposed model is made predictive by employing group contribution methods to estimate the pure compound input parameters required to perform thermodynamic calculations with the model. These parameters can be calculated for closed-shell molecules, free radicals, and transition states, with an average deviation of less than 10% with respect to the benchmark database containing experimental data as well as data obtained from quantum-based calculations and QSPR-type correlations. The *tc*-PR/COSMO-RS model is able to predict the solvation free energies of activation for H-abstraction reactions with an accuracy of approximately 0.2 kcal/mol, offering a high-throughput and accurate solution for integrating solvation effects into detailed kinetic models in the liquid phase.

## 1 Introduction

Detailed kinetic models are valuable tools for understanding and predicting complex reactions, including combustion and pyrolysis,^1^ liquid-phase oxidation,^2^ polymerization,^3^ and atmospheric chemistry.^4^ Developing a detailed kinetic model involves breaking down complex reactions into elementary steps, providing a clear understanding of the underlying reaction pathways. Once a detailed kinetic model is established, identifying its key pathways is crucial for several applications, including the design and optimization of chemical reactors, flow diagnostics with computational fluid dynamics (CFD), and scaling up from laboratory to industrial processes. Detailed reaction mechanisms are often developed by employing the so-called kinetic generators, such as EXGAS,^5^ RMG,^6^ MAMOX,^7^ NetGen,^8^ and GENESYS.^9^ This sort of tool combines extensive databases containing predefined reaction rate rules derived from experimental data, theoretical calculations, and group additivity methods to generate the required thermo-kinetic properties.^10^ The thermodynamic and kinetic data produced by kinetic generators are primarily developed for gas-phase applications. However, in certain cases, there is an interest in using these mechanism generators in phases other than gases. For instance, liquid-phase oxidation takes place in the process aimed to produce phenol and KA oil (two important precursors in the polymer sector^11,12^) or in the Hock process to yield phenol.^13,14^ The aging, i.e., liquid-phase oxidation of liquid fuels,^15^ lubricants,^16^ oil paint,^17^ and vegetable oils^18^ are also applications that would benefit from a better understanding of kinetics phenomena through the use of detailed kinetic models.

Nonetheless, converting the gas-phase thermo-kinetic data provided by automatic kinetic generators into liquid-phase data to predict kinetic mechanisms in the liquid phase poses an additional challenge due to the solvent dependency of liquid data.^19^ Jalan et al.^20^ proposed a method to bridge the gap between gas and liquid phases, by adding solvation corrections to the gas-phase data. This can be done because the solvation process itself represents the change in a thermodynamic property when a molecule is transferred from the perfect gas state into a real solvent.^21^ It can be noticed that solvation properties fundamentally reflect the probability and strength of the interactions present in liquid phases. Liquid-phase interactions include contributions from a range of intermolecular interactions between solute and solvent molecules, including electrostatic, van der Waals, and hydrogen bonding interactions.^22^ For including the solvation process in the calculation of intrinsic reaction rates in liquids () based on the perfect gas condition (*k*_gas_), the following equation can be used.
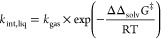
1where ΔΔ_solv_*G*^‡^ stands for the solvation free energy of activation of a given reaction, i.e., the difference in solvation free energy (Δ_solv_*g̅*_*i*_) between its transition state structure (TS) and its reactants,^20^ as denoted by eq 2. For liquids far from the critical point, for which the incompressible fluid assumption is valid, the solvation quantities become functions of the temperature (*T*) and composition of the system (accounted for by the molar fraction vector *z*).
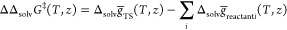
2The solvation free energy (Δ_solv_*g̅*_*i*_) of a given species “*i*”—also named the solvation chemical potential of *i*—in a liquid solvent can be estimated from different models or techniques available in the literature,^23^ such as molecular dynamics, linear solvation energy relationships, machine learning models,^24−26^ continuum solvation models,^27−29^ and equations of state.^30−32^ These methods have been extensively studied for predicting solvation properties, but their development is generally limited to closed-shell solutes, i.e., molecules with fully filled electron orbitals and stable electronic configurations. To address open-shell molecules (species with unpaired electrons, such as free radicals), we previously formulated a predictive model based on the group contribution (GC) concept that combines the Peng–Robinson cubic equation of state with a continuum solvation model called COSMO-RS (Conductor-like Screening Model for Real Solvation).^33^ In the present study, we aim to apply a similar methodology to analyze transition states, with a focus on hydrogen abstraction reactions, which are foundational steps in any oxidation mechanism. In short, these reactions are characterized by the removal of a hydrogen atom from a molecule (R–H) by a free radical (X^·^), as illustrated below.
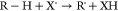
3To enable the determination of ΔΔ_solv_*G*^‡^ to be used in the correction of H-abstraction reaction rates from the gas phase to liquid solvents, this work will refine and extend the GC-based equation of state previously applied to closed-shell solutes and free radicals, now incorporating transition states.^34^

## 2 Modeling

### 2.1 Equations of State as a Universal Approach To Compute Solvation Data

Equations of state (EoSs) are versatile thermodynamic models that mathematically relate pressure, temperature, molar volume, and composition. Inspired by van der Waals' pioneering work in the 19th century, they have become the cornerstone of thermodynamic models, providing a comprehensive description of the behavior of pure compounds and mixtures across fluidic states of matter, including gases, liquids, and supercritical fluids. These models have been extensively employed due to their good trade-off between accuracy and computational costs.^35^

The success of the EoS models lies in their ability to accurately estimate a wide range of properties, including energetic and phase equilibrium properties.^36^ When they are combined with the entropy-scaling concept, these models can also be used to predict transport properties (shear viscosity, thermal conductivity, and self-diffusion coefficient). In the context of kinetic modeling, Moine et al.^30,37,38^ demonstrated, for the first time in the literature, that EoSs can also be used to calculate solvation quantities. After some mathematical development, they arrived at a relationship between the solvation free energy (Δ_solv_*g̅*_*i*_) of a solute *i* in a solvent and variables that are straightforwardly calculated by a cubic EoS, namely, the molar volume of the solvent phase (*V*_liq_) and the fugacity coefficient of solute *i* in solution (φ_*i*,liq_). The relationship between these quantities and the solvation free energies is given by eq 4. These variables are functions of the temperature (*T*), pressure (*P*), and molar composition (*z*) of the system.
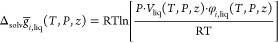
4In their benchmark study, Moine et al.^30^ employed a predictive version of the Peng–Robinson cubic EoS to calculate Δ_solv_*g̅*_*i*_ of infinitely diluted solutes *i* in pure liquids. The main advantage of using a cubic EoS is the straightforward parametrization compared to other, more complex equations of state. Cubic EoSs require a small number of input parameters that are often readily available or can be estimated from limited experimental data or predictive models. This makes this sort of model a suitable approach for obtaining predictions of solvation free energies within detailed kinetic models, as it can be applicable to a wide range of chemical species and also be extended to unconventional solutes, like free radicals and transition states. Moreover, it has been demonstrated that the cubic EoS approach can provide predictions of solvation data with a comparable level of accuracy to that of continuum solvation models,^31^ such as the COSMO-RS implementation in the COSMOTherm software, which is commonly used to predict solvation energies.

In this regard, the following sections present a description of the parametrization process of cubic EoS (by “parameterization,” it is meant the definition of all the input parameters needed to perform thermodynamic calculations with an EoS), together with an account of how it can be adapted to transient molecules through a predictive approach. To this end, we will base ourselves on the *translated-consistent* (tc) Peng–Robinson equation of state (*tc*-PR EoS),^39,40^ which integrates all the latest advances of the cubic EoS family. The *tc*-PR EoS is presented in detail in the next section.

### 2.2 Translated-Consistent Peng–Robinson Equation of State (*tc*-PR EoS)

Among all versions of the cubic family of EoSs, the *tc*-PR model is one of the most accurate options when it comes to predicting the thermodynamic properties of pure compounds, such as vapor pressures, liquid densities, vaporization enthalpies, and heat capacities. In regard to this topic, Pina-Martinez et al.^40^ obtained mean relative deviations below 5% for most of pure component properties with respect to a database comprising 1,800 molecules. This good performance justifies the choice of this cubic EoS as our base model. The formulation of the *tc*-PR EoS for a pure component *i* is given in eq 6. As with any translated version of cubic equations of state, the *tc*-PR EoS has three parameters: the attractive parameter (*a*_i_), the covolume of the nontranslated EoS (*b*_i_), and a constant correction called *volume translation* (*c*_i_).
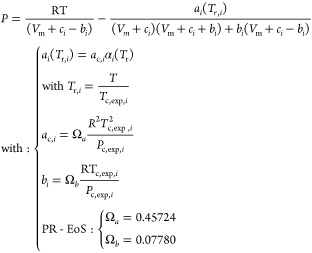
5where , , and  are the pressure, temperature, and molar volume of a fluid, respectively. The parameter  is related to the attractive forces between molecules, the parameter  is the volume occupied by one mole of the substance at infinite pressure (when the EoS is not translated, i.e., when *c*_*i*_ = 0), and *c*_*i*_ has the role of improving the calculation of volumetric properties of liquids.^41,42^ In eq 5, the parameter  corresponds to the attractive parameter when the reduced temperature  is equal to one, i.e., at the experimental critical temperature (). As previously mentioned, the *tc*-PR EoS is described as “translated” due to the inclusion of the translation parameter (). This parameter adjusts the molar volume derived from the original cubic EoS formulation (where the  parameter is not considered), by effectively translating it by a constant value *c*_*i*_, as highlighted in eq 7.
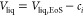
6where  is the final molar volume, and  is the molar volume obtained by solving the cubic EoS without accounting for the volume translation parameter. This constant correction on  is often referred to as “Volume translation of Peneloux et al.”,^41^ and its goal is to correct systematic errors in the prediction of liquid molar volumes that arise from the original formulation of a cubic EoS (see Figure 1 of ref (42)). The parameter  is determined in order that the equation of state reproduces the experimental molar volume of a pure liquid when the reduced temperature reaches 0.8. This value can be fitted to experimental data or predicted via generalized correlations based on critical constants and acentric factors , as the one given in eq 8.^43,44^
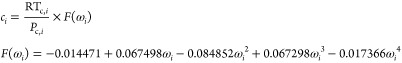
7The *tc*-PR EoS is also said to be “consistent” due to the use of a thermodynamically consistent α-function used to describe the temperature dependence of the attractive parameter *a*_*i*_(*T*). Le Guennec et al.^45,46^ tested the consistency of 12 widespread α-functions. They concluded that eight or more than 12 of these α-functions failed in passing the consistency tests. Among the remaining ones, the three-parameter Twu-91 α-function was the one with the best trade-off between complexity (small number of parameters) and flexibility, the reason why it was chosen to be used in the *tc*-PR model. The formulation of the Twu-91 α-function is given in eq 9.
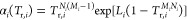
8where *L*_*i*_, *M*_*i*_, and *N*_*i*_ are pure compound parameters fitted to experimental data, such as vapor pressures and liquid densities. The Twu-91 α-function parameters (along with volumetric translation constants) can be found elsewhere for 1800 pure compounds.^40^ For other chemical species, the following generalized correlations based on acentric factors (ω_*i*_) can be used instead:^45^
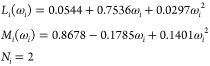
9

### 2.3 From Pure Compound to Mixtures through Mixing Rules

Above, we presented the *tc*-PR EoS applied to pure components. In mixtures, the formulation presented in eq 5 remains the same, but the parameters , , and  are specific to the mixture considered (1-fluid theory). The calculation of these mixture parameters involves the knowledge of the corresponding parameters associated with each component of the mixture^47^ and the composition of the system in terms of molar fractions (vector of molar fractions denoted ). These relations, introduced in eqs 10–12, are known as *advanced mixing rules* and are derived from the work of Huron and Vidal.^48^
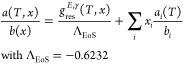
10
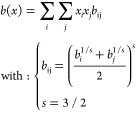
11
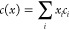
12The advanced mixing rules were developed with the aim of extending the applicability of cubic EoSs to complex systems involving polar compounds,^49^ which was a loophole for the so-called *classical mixing rules* first proposed by van der Waals.^50^ The key insight of the advanced mixing rules is that the attractive parameter (the one related to the energies of interaction between molecules) includes an excess Gibbs energy term  for describing the deviation of a real solution from ideal behavior. This deviation arises from differences in size, shape, and interactions between molecules of different species in a solution so that the inclusion of an excess Gibbs energy term can capture all these effects.

In the mixing rule presented in eq 10, only the residual part of the excess Gibbs energy  is required. The residual part (also called the enthalpic contribution) reflects differences in terms of interactions between the different species of the mixture. The entropic or combinatorial part is not retained in the formulation of the mixing rule presented in eq 10. The calculation of  can be done based on the composition of the system (*x*) and the estimation of the residual contribution to the activity coefficients  of the molecules in the mixture, as detailed in eq 13.
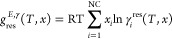
13The major advantage of this approach is that activity coefficient models are highly effective for describing nonideality effects in the liquid phase. A variety of options are available: from simple, purely correlative models to more sophisticated models based on statistical thermodynamics. The most accurate models are typically those relying on robust theories, such as the local composition^51,52^ and quasi-chemical solution theories,^53^ which include the Wilson,^54^ NRTL,^51^ and UNIQUAC^55^ models. In addition to the theoretical background, these models often yield accurate results because they rely on binary interaction parameters (BIPs) fitted to experimental data.^47^ Predictive models, such as UNIFAC^56^ (the predictive version of UNIQUAC), can circumvent this dependence on experimental data, making the model more flexible. The UNIFAC model is based on the description of molecular structures and their interactions with functional groups. Many well-known EoSs make use of the UNIFAC model, like PSRK,^57−59^ VT-PR,^60^ and UMR-PRU.^61^ Despite being a more comprehensive model, UNIFAC has a significant limitation: it requires binary interaction parameters (BIPs) fitted from an extensive and diverse experimental database to describe the interactions between functional groups. This necessity makes it challenging to introduce new groups, as it demands fitting a large set of new BIPs values, posing a considerable obstacle to its adaptability to treat molecules with no experimental information in the liquid phase.

Other types of continuum solvation model that do not suffer from these limitations could be considered, such as computational chemistry models, e.g., SMD,^62,63^ PCM,^64^ and COSMO.^65,66^ These models allow macroscopic properties to be calculated from quantum chemistry methods and continuum solvation theories.^67^ The significant advantage of this approach is that it does not require the direct use of experimental data for its implementation. Instead, it relies on the knowledge of the atomic coordinates and electronic structure obtained from quantum calculations to model solvation effects and predict solvent/solute interactions. This quantum-based methodology allows for a more flexible analysis that can be extended to any molecule with known atomic coordinates.

**Figure 1:**
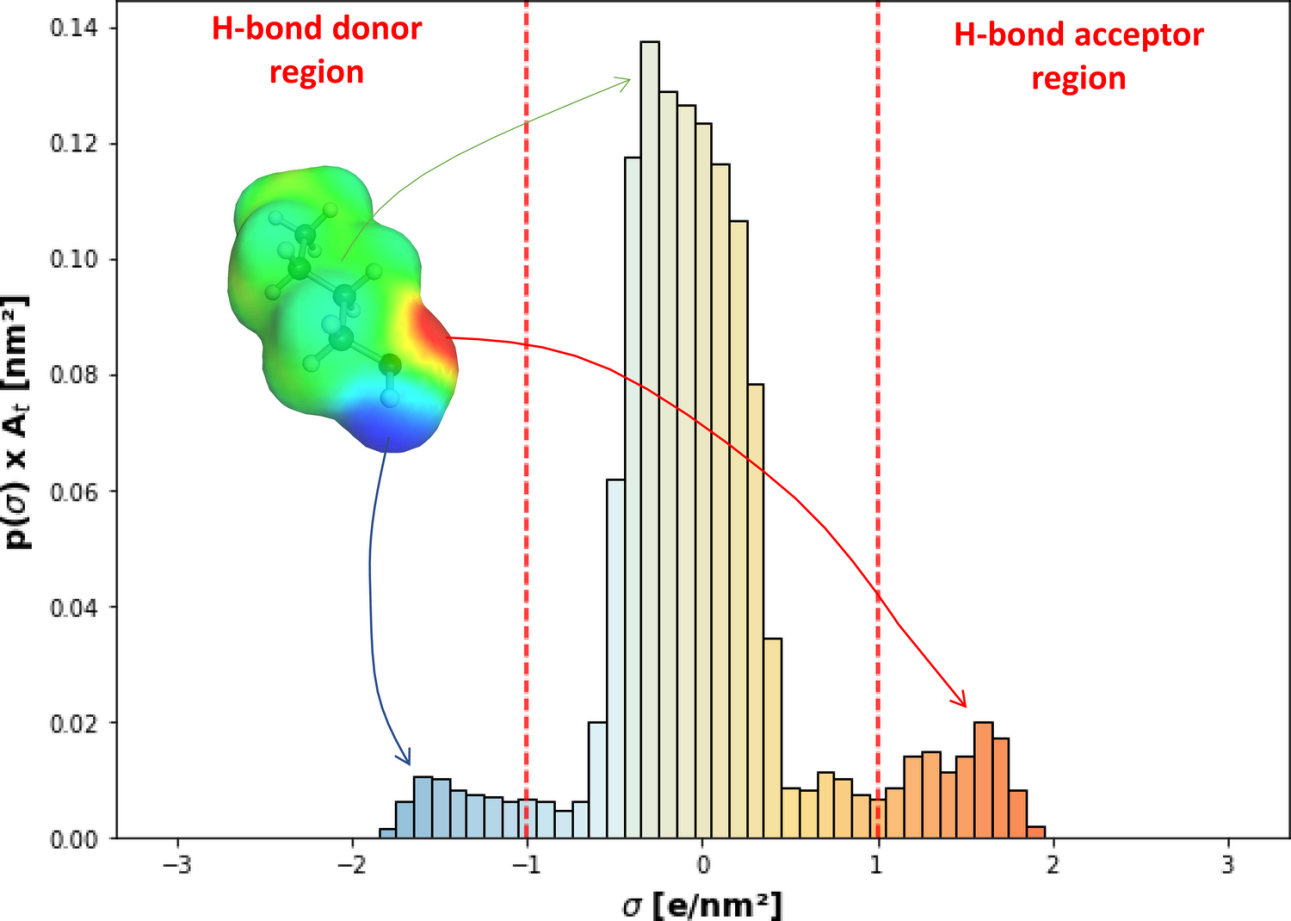
Charge density distribution (or σ -profile) of the surface segments of the 1-butanol COSMO cavity extracted from the COSMObase23 (BP-TZVPD-FINE level of theory). In the figure, *p*(σ) is the normalized charge density distribution, and *A*_t_ is the total area of the molecular cavity.

Among the different options, COSMO-RS and its variants represent the most widely used continuum solvation models and have been a topic of great interest in the chemical industry in the last years.^68^ The success of COSMO-based models arises from their rigorous application of statistical thermodynamics principles, equivalent to Guggenheim’s quasi-chemical theory used with UNIFAC functional groups, but applied to interacting surface segments derived from quantum calculations. The main information captured from these quantum calculations is the distribution of charge densities of surface segments (σ) on a molecular cavity when it is placed in a virtual conductor with infinite dielectric constant. The distribution of σ values is presented in a histogram called the σ-profile. An example of the discretized σ-profile is given in Figure 1. For more detailed information on σ-profiles, readers are encouraged to refer to Andreas Klamt’s book, which offers an in-depth exploration of this subject.^69^

Broadly speaking, COSMO-RS describes how different surface segments of a molecule interact with the segments of its surroundings, providing data for predicting solvation and mixture behavior based on this very localized molecular information. This eliminates the need for binary interaction parameters (BIPs) in COSMO-based models, making it a more flexible tool than UNIFAC.

### 2.4 COSMO-RS as a Flexible *g*^*E*^ Model within Mixing Rules

Given the predictive capabilities of COSMO-based models, they become an interesting option to be incorporated as an activity coefficient model into the mixing rules of cubic EoS (term  in eq 10). This combination has already been the subject of our previous studies investigating the calculation of solvation properties^32,33^ and will also be applied in this work. As such, we will discuss below the formulation of this model, basing ourselves on the version of COSMO-RS published by Klamt and Eckert in 2000.^70^ In this version of COSMO-RS, a residual activity coefficient () is calculated from eq 14.
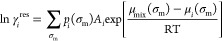
14where  is the σ-profile of solute *i*, and  stands for its molecular cavity surface. Moreover,  and  are the so-called σ-potentials, which represent the change in the chemical potential of a specific surface segment () when it is transferred from the reference state (the molecule embedded by the virtual conductor) to the mixture and to the pure compound *i*, respectively. The implicit eq 15 is used to calculate the σ-potentials.
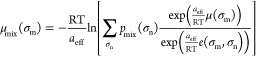
15where *a*_eff_ is the effective contact area between two segments m and n, and  is the contact energy of these two segments. The normalized σ-profile of the mixture () can be expressed as a linear combination of the σ-profiles of pure compounds and their respective molar fractions in the mixture , as shown in eq 16.
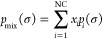
16It should be noted that in eq 15, the calculation of the σ-potential in the mixture is shown (). To calculate it in a pure compound *i* (), it is necessary to use the pure compound σ-profile (). Also, it should be noted that in this version of COSMO-RS, the contact energy () only includes electrostatic (or misfit) and hydrogen bond interactions, which are calculated by eq 17. Dispersive interactions are not considered because it is assumed that they are the same in both the liquid and reference states (molecules embedded in the ideal conductor). Thus, they do not contribute to the activities.
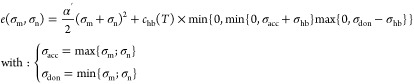
17In the contact energy equation, α′ is the misfit interaction parameter,  is a cutoff charge density value for hydrogen bonding, and  is the hydrogen-bonding interaction parameter, the latter being a function of temperature, which can be described by eq 18.^69^ This function refers to an empirical model that incorporates the effect of the temperature on hydrogen bonding.
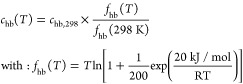
18COSMO-RS treats hydrogen bonds as nonspecific interactions, meaning it does not differentiate between various types of hydrogen bonds (e.g., O–H···O vs N–H···O). As a result, the same set of universal constants is used for all types of hydrogen bond interactions. This simplified way of describing hydrogen bonds, along with the omission of the dispersion term, serves to reduce the number of universal parameters, thereby limiting the complexity of the model.

In this study, all COSMO-RS universal constants were extracted from a previous study,^32^ and their values are given in Table 1. It should be noted that that these constants are universal because they are applicable to all systems modeled by this version of COSMO-RS, regardless of their constituents. In the aforementioned study,^32^ these universal constants were fine-tuned on experimental values of solvation data.

**Table 1:** COSMO-RS Parameters for the BP-TZVPD-FINE Level of Theory^32^

parameter	unit	final value
*a*_eff_	A^2^	4.73
α′	kJ/mol/A^2^	10,473
*c*_hb_	kJ/mol/nm^2^	48,321
σ_hb_	e/A^2^	0.011

### 2.5 Establishing a Predictive Equation of State Based on the Group Contribution Concept

Although COSMO-RS increases the predictive power of the cubic EoSs, since it only relies on σ-profiles obtained from quantum calculations, it remains a semipredictive model. This is because the pure compound parameters , , and  are usually calculated from experimental values of critical temperatures (), critical pressures (), and acentric factors (). Nonetheless, when dealing with transient molecules, such as free radicals and transition states, acquiring the necessary inputs for calculating those parameters poses significant challenges due to the inexistence of experimental data. Thus, predictive methods become indispensable tools in such cases.

For this matter, in our previous study,^33^ we developed simplified and easy-to-use first-order group contribution (GC) methods for addressing these challenges, allowing a predictive estimation of the Peng–Robinson EoS parameters. Specifically designed to describe closed-shell molecules and C/H/O free radicals, these GC methods offer a robust framework for predictive analysis in cases in which there is a lack of experimental data. A step-by-step guide of the proposed GC-based approach to the EoS is given below.

1. and : Calculation of the attractive parameter at the critical temperature () and the covolume of the untranslated EoS (), both by group contribution methods through eqs 19 and 20. In these equations,  is the occurrence of the group  in the molecule, whereas  and  are the contributions of the functional group  in the prediction of  and . It should be noted that  and are universal constants given in Table 2.
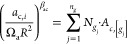
19
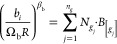
20
2. (intermediate parameter): Calculation of the attractive parameter at the limit when  from a group contribution approach, using eq 21, in which  is a universal constant (also given in Table 2), and  is the contribution of the group  in the prediction of . To simplify the parametrization process, the α-function proposed by Soave^71,72^ is considered for the molecules parametrized via group contribution (and not the Twu-91 α-function, which is yet the preferred alpha function in the *tc*-PR equation), as it corresponds to a simpler α-function that requires only one compound-specific parameter.
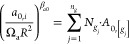
21
3. α**_*i*_**(*T*): The GC-predictions of *a*_c_ and *a*_0_ are used to determine the compound-specific parameter *m*_*i*_ of the Soave α-function. The GC-predictions of  and  are then used to determine the critical temperature (). Obtaining  and  allows the Soave α-function (and therefore the attractive parameter, ) to be calculated at any temperature .
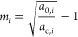
22
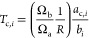
23
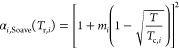
24
4.**:** If the solute *i* is highly diluted in the mixture, the volume translation parameter can be disregarded (). Otherwise,  can be obtained via generalized correlations (see eq 7). For this purpose,  and  must be estimated. The acentric factor  can be calculated from the correlation developed in 1976 by Peng and Robinson^73^ to estimate  from the knowledge of  by solving eq 25, while  can be simply obtained by combining  and , as shown in eq 26.
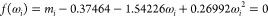
25
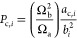
26
5.: Calculation of σ-profiles from the group contribution concept to avoid the need for time-consuming quantum mechanical calculations. This is done using eq 27.
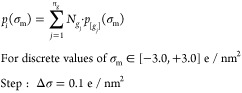
27

**Table 2:** Universal Constants That Appear in the Proposed GC-Based Models^33^

parameter	value
β_ac_	0.67
β_b_	0.80
β_a0_	0.48

The Peng–Robinson EoS parametrization approach showed above was developed for closed-shell molecules and free radicals. We now present an extension of this approach to transition states. Broadly speaking, this extension is achieved by adding new functional groups that represent transition state structures to the previously developed GC methods. We will also discuss the methods used to fit the group contribution parameters of functional groups [*g*] (that are *A*_c,[*g*]_, *A*_0,[*g*]_, *B*_[*g*]_, and *P*_[*g*]_(σ_m_)).

## 3 Methodology

### 3.1 Decomposition of Molecules, Free Radicals, and Transition States into Groups

First, we present a revised group decomposition scheme that now involves transition states. This scheme follows the same reasoning as that formulated in earlier work on molecules and radicals,^33^ but with a few modifications. In our previous work, free radical group corrections were introduced based on the assumption that the property values of the parent molecules (i.e., the closed-shell molecule with a “H” instead of the free electron of the radical) could be corrected by the addition of a radical group contribution, which was calculated from experimental data or quantum-based results. In this version, we will not treat radicals on the basis of the parent molecule, but we will treat them independently. The free radical functional groups are no longer defined as corrections to the parent molecule properties, but rather as independent group contributions. A similar concept will be applied to transition states, where their reactive moieties are defined as groups. The new framework is illustrated by the examples given in Table 3, which shows the method for decomposing closed-shell molecules, free radicals, and transition states into groups. All available groups are given in the Supporting Information file.

**Table 3:**
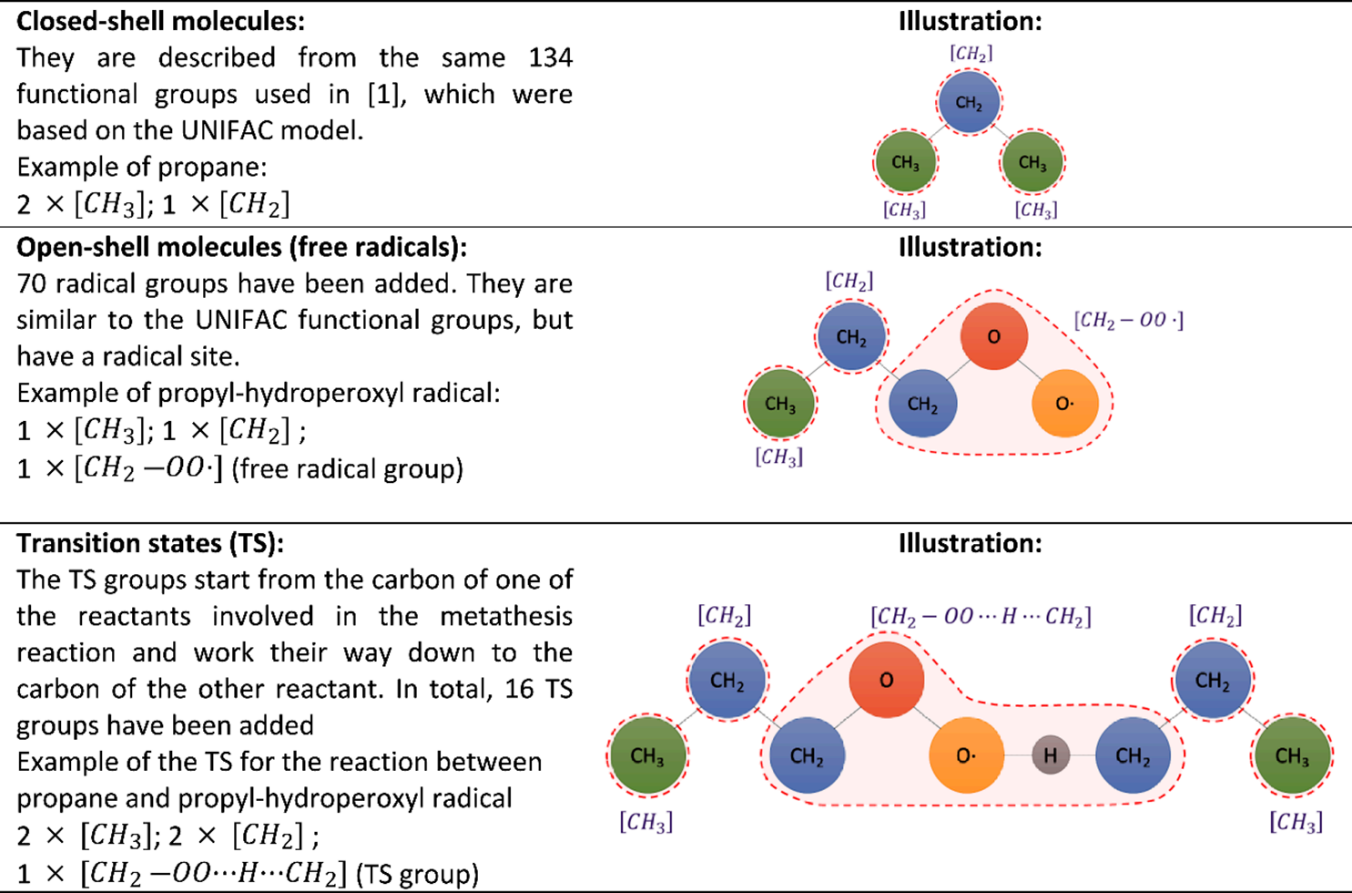
Decomposition Scheme of Molecules, Free Radicals, and Transition States into Groups

### 3.2 Generating a Reference Database for Parameter Identification

In order to estimate the contribution parameters (*A*_*c*,[*g*]_, *A*_0,[*g*]_, *B*_[*g*]_, and *P*_[*g*]_(σ_m_)) of each functional group, the first step is to develop a reference database containing the input parameters of the PR/COSMO-RS EoS (*a*_c,*i*_, *a*_0,*i*_, *b*_*i*_, and *p*_*i*_(σ)) of molecules, free radicals, and transition states. These data are used for fitting the contribution values of each functional group.

#### 3.2.1 COSMO Files and σ-Profiles of Molecules and Radicals

Starting with the inputs for the COSMO-RS model, a reference database of 1,600 σ-profiles ((σ)) of closed-shell molecules was created based on COSMO files provided by the software *COSMOTherm* v.23. Additionally, COSMO files and the corresponding σ-profiles for 126 C/H/O free radicals were generated using Turbomole v.23 at the same level of theory (BP-TZVPD-FINE). In total, 12 different types of free radicals were considered, according to Table 4.

**Table 4:** Types of C/H/O Free Radicals Considered in This Study

radical atom	type of radical	description
carbon centered radical	primary (RCH_2_·)	one carbon atom attached to the radical carbon with *sp*^*3*^ hybridization
	secondary (RCH·R′)	two carbon atoms attached to the radical carbon with *sp*^*3*^ hybridization
	tertiary (RC·(R ′)_2_)	three carbon atoms attached to the radical carbon with *sp*^*3*^ hybridization
	vinyl (RC=CH·)	unpaired electron on an *sp*^*2*^ carbon atom that is part of a double bond with another carbon
	acetylenic (RC≡C·)	unpaired electron on an *sp* carbon atom that is part of a triple bond with another carbon.
	aryl (Ar·)	unpaired electron on one of the carbon atoms of an aromatic ring.
	benzyl (Ar–CH·R)	unpaired electron on the carbon atom of an alkyl group attached to an aromatic ring
	acyl (RC·O)	unpaired electron on a carbon atom from a carbonyl group
oxygen centered radical	acyloxyl (R·CO)	unpaired electron on an oxygen atom from a carbonyl group
	hydroxyl (O·)	oxygen atom with an unpaired electron
	alkoxyl (RO·)	derived from an alcohol. The hydrogen atom from the hydroxyl group is removed
	peroxyl (ROO·)	characterized by an oxygen–oxygen bond with an unpaired electron attached to a peroxyl group

In addition to the data for closed-shell molecules and free radicals, COSMO files and their corresponding σ-profiles were generated for 80 transition states involved in H-abstraction reactions. These transition states are associated with reactions where an alkoxyl or peroxyl radical (RO or ROO) abstracts a H atom from linear or branched alkanes (R–H), as exemplified below.
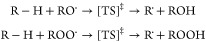
28The list of reactants considered is shown in Table 5. The 80 reactions (and transition states) considered are therefore obtained from 80 different combinations of these reactants. These combinations make it possible to evaluate the effect of chain size and branching.

**Table 5:**
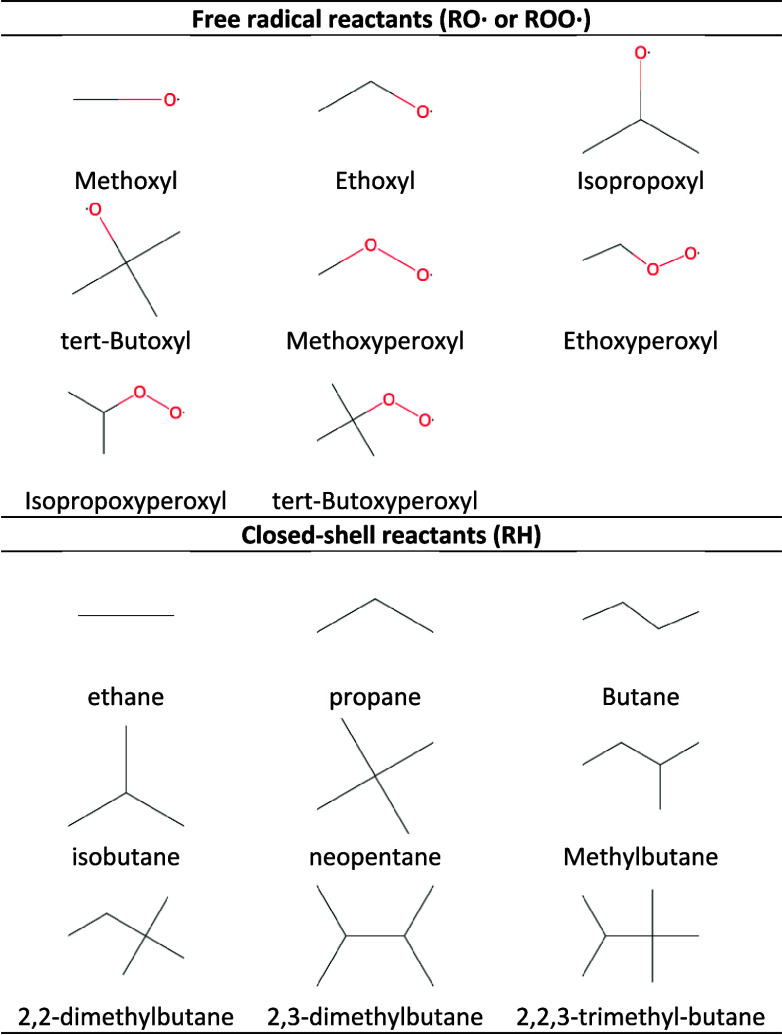
Reactants Considered for the Formulation of Hydrogen Abstraction Reactions

#### 3.2.2 COSMO Files and σ-Profiles of Transition States (TS)

The development of a database of COSMO input files for transition states is not as straightforward as that for molecules and radicals. TSs are first-order saddle points on the potential energy surface (PES), and optimizing their geometry is more difficult than for species like molecules or radicals, for which the stable state is associated with a minimum of the PES. In particular, we need to verify that the convergence of the geometry leads to a first-order saddle point by ensuring that the Hessian matrix has a single negative eigenvalue (one imaginary frequency corresponding to the reaction coordinate). This requires additional vibrational frequency calculations, compared with the template method implemented in Turbomole, which generates COSMOTherm input files only for minima of the PES.

Moreover, the molecular structures of the TSs considered in this work have a large number of possible conformations, and the influence of multiconformers on the computed solvation Gibbs energies of TSs remains unknown in the literature. In a previous study,^33^ we compared the impact on the Gibbs solvation energies of using a single (more stable) or multiple conformers in the COSMOtherm calculation. This comparison was carried out over 67 000 experimental data for binary systems, and it was shown that the mean unsigned error remains nearly identical (0.33 kcal/mol) when using the single optimal conformer, i.e., the one having the lowest energy, and all conformers (0.29 kcal/mol). As the TSs have looser geometries, the question of the influence of conformers on the calculation of  with COSMOTherm was first explored for the reactions RH + RO^·^ → R^·^ + ROH.

The geometries and vibrational frequencies calculations of the TSs were performed with the Molpro software version 2022.1^74,75^ at the BP/def1-TZVP^76^ level of theory with density fitting. Calculations were performed in the vacuum (gas phase) using the COSMO model implemented in Molpro. Comparisons with known molecules have shown that solvation Gibbs energies calculated with COSMOTherm, using inputs generated with Molpro or Turbomole, gave identical results. After the calculation, we systematically checked that all of the transition states obtained had only one imaginary frequency. This computational method corresponds to the “TZVP” parametrization of COSMOTherm. Since the “TZVP-FINE” parametrization was used for radicals and molecules, additional calculations were performed to match this parameter set. Single-point energy calculations at the BP/def2-TZVPD level of theory were performed on the fixed gas and COSMO geometries of TS obtained with Molpro, using the Turbomole software because the FINE cavity construction algorithm is not available in Molpro. The dual-level calculation, i.e., the geometry optimization at the BP/TZVP level and the BP/def2-TZVPD single-point energy, with the FINE cavity construction algorithm, corresponds to the standard “TZVP-FINE” method in COSMOTherm. The computed TZVP-FINE gas-phase energy was included in the COSMOTherm input.

**Figure 2:**
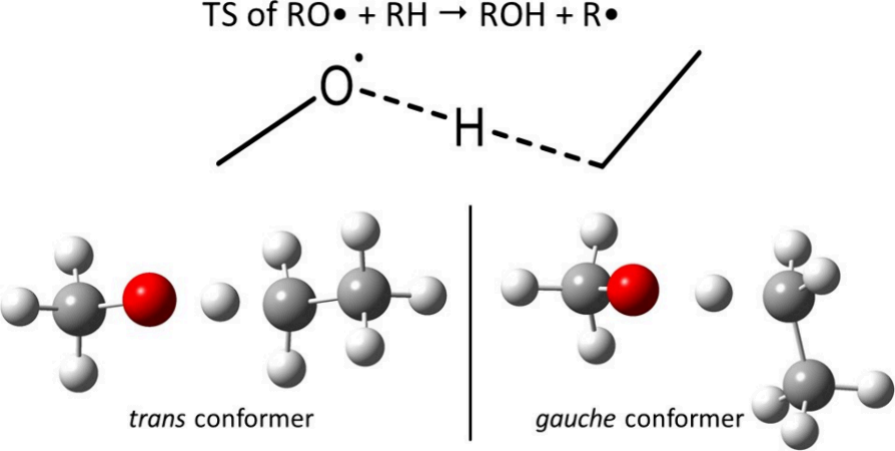
2D and 3D representations of the minimal TS structure of the RH + RO^·^ H-abstraction when RH is an alkane (TS1 below).

The TS database for RH + RO^·^ → R^·^ + ROH was built, based on the minimal TS structure for this type of reaction, by considering alkanes for the RH molecule. Figure 2 presents the transition state corresponding to this H-abstraction.

**Figure 3:**
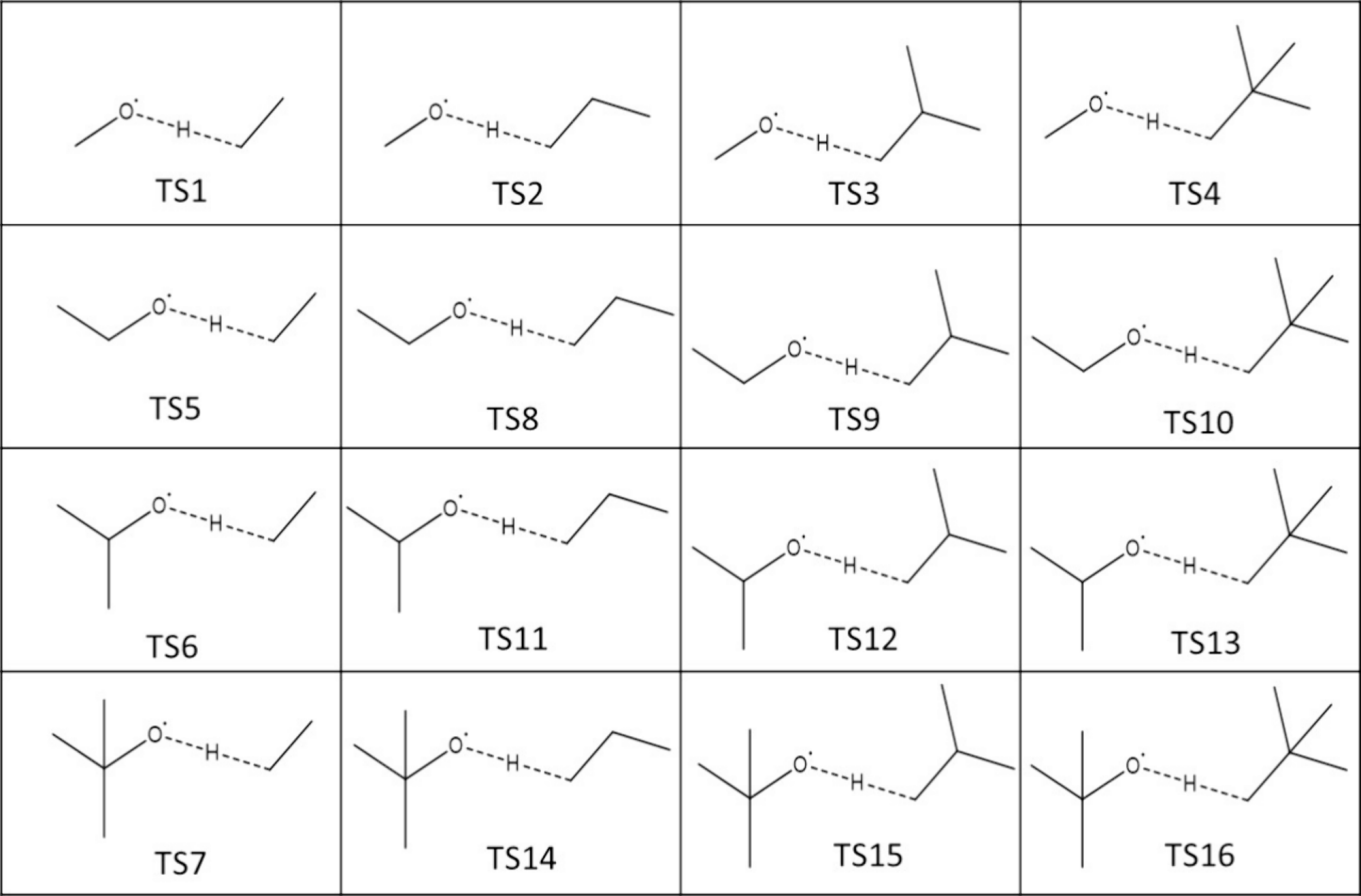
TS structures obtained from the different substitutions of H atoms by CH_3_ in TS1.

For this minimal TS structure, two conformations were optimized at the BP/TZVP level, named *trans* and *gauche*. In order to create other TSs for enriching our database, we started from these two conformers and generated new TSs by considering all possible combinations of substitution of a hydrogen atom by a methyl group. This led to 16 different TSs (presented in Figure 3, with several conformers identified for each of them.

The combinations presented in Figure 3 all belong to the same reaction class: H-abstraction by RO^·^ of a primary H atom. In other words, the reacting moiety defined as a group remains unchanged in all 16 TSs. The first row (TS1 to TS4) shows the increase of the size of the alkane from which the H atom is abstracted, while the size of the RO^·^ radical remains constant. The first column (TS1 to TS7) presents the exact opposite, and TS8 to TS16 results from all the remaining combinations.

**Figure 4:**
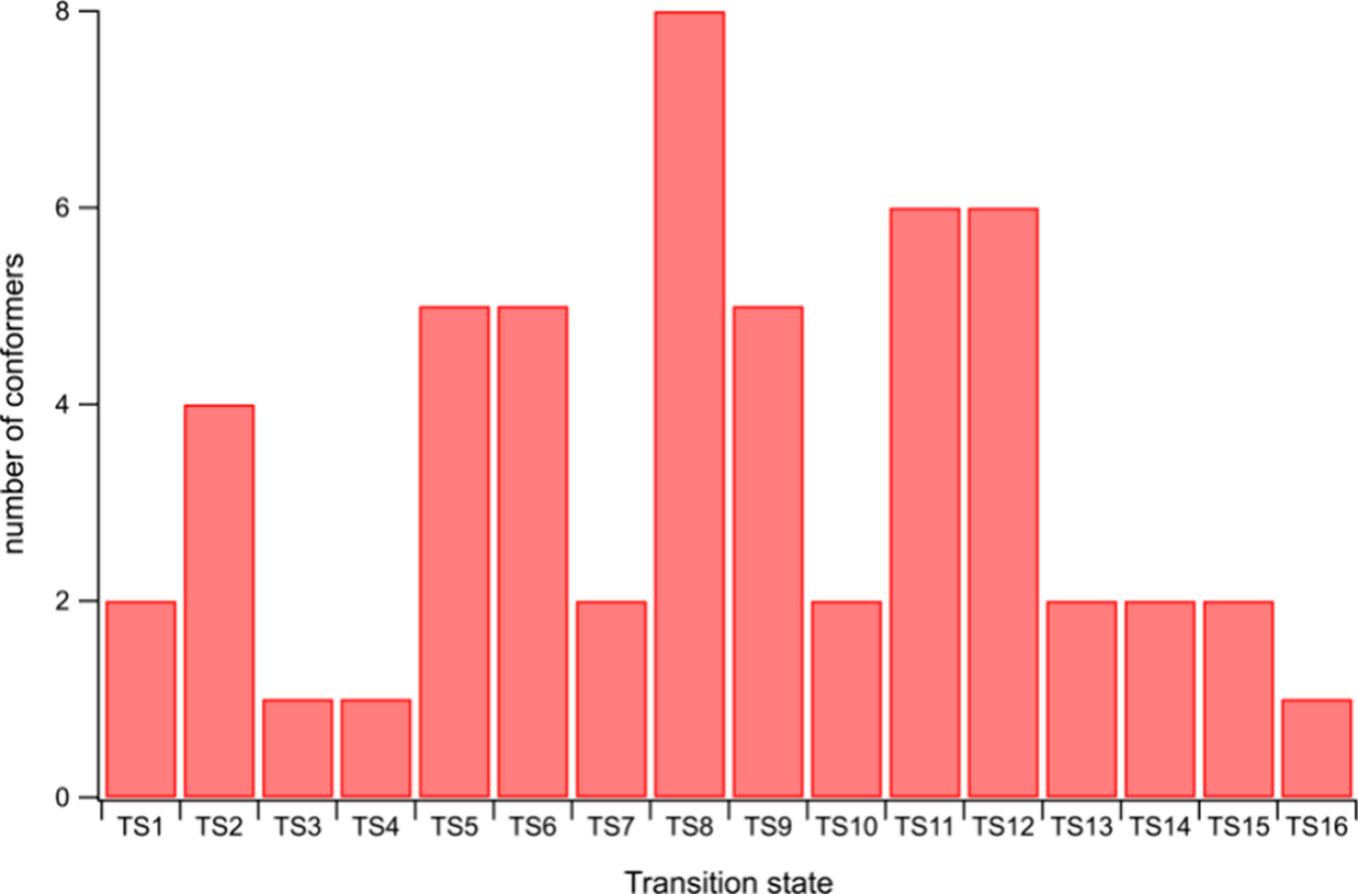
Number of conformers optimized at the BP/TZVP level in the COSMO cavity for each TS of Figure 3.

Once done, we generated all the possible conformers for each of these 16 TSs. Finally, for the 16 TSs presented above, a total of 54 conformers were optimized. Their distribution is given in Figure 4.

It can be observed that the number of conformers is lower for the TS structures involving a quaternary carbon. On the basis of these identified conformers, it is possible to compare the effect on the calculated chemical potentials of solvation Δ_solv_*g̅* of the TS by including all conformers in the COSMO-RS calculation or using only the lowest energy, gas-phase conformer. The computed *COSMOTherm* v.23 results in the *n*-decane solvent are presented in Table 6.

**Table 6:** Comparison between the Chemical Potential of Solvation () of TSs in *n*-Decane, Computed Using Only the Lowest-Energy Conformer (min) and the Conformation Equilibration Scheme in COSMOtherm (All)^a^

	number of conformers	mean unsigned error (kcal/mol)
TS1	2	0.04
TS2	4	0.06
TS3	1	N/A
TS4	1	N/A
TS5	5	0.08
TS6	5	0.06
TS7	2	0.08
TS8	8	0.17
TS9	5	0.02
TS10	2	0.02
TS11	6	0.17
TS12	6	0.07
TS13	2	0.13
TS14	2	0.05
TS15	2	0.01
TS16	1	N/A
MUE (min vs all conformers)		0.1kcal/mol
MPE (min vs all conformers)		–1%

a MUE stands for ‘Mean Unsigned Error”, while MPE stands for ‘Mean Percentage Error’.

The deviation between the use of all the conformers and the use of the lowest-energy conformer identified in the gas phase to compute  of TSs remains very small on average. Trends do not seem to be drawn as a function of the number of conformers, as, e.g., the absolute deviation for TS8 with eight conformers is similar to that for TS13 with two conformers. Changing the solvent from *n*-decane to water does not affect these results as the MUE (as defined in Table 6) remains identical. On the basis of these comparisons, whose results are consistent with those obtained previously on molecules,^33^ it can be concluded that the use of the single lowest-energy TS conformer in the gas phase leads to sufficiently accurate Gibbs energies of solvation. In the subsequent part of this work, only the lowest conformation has been used.

**Figure 5:**
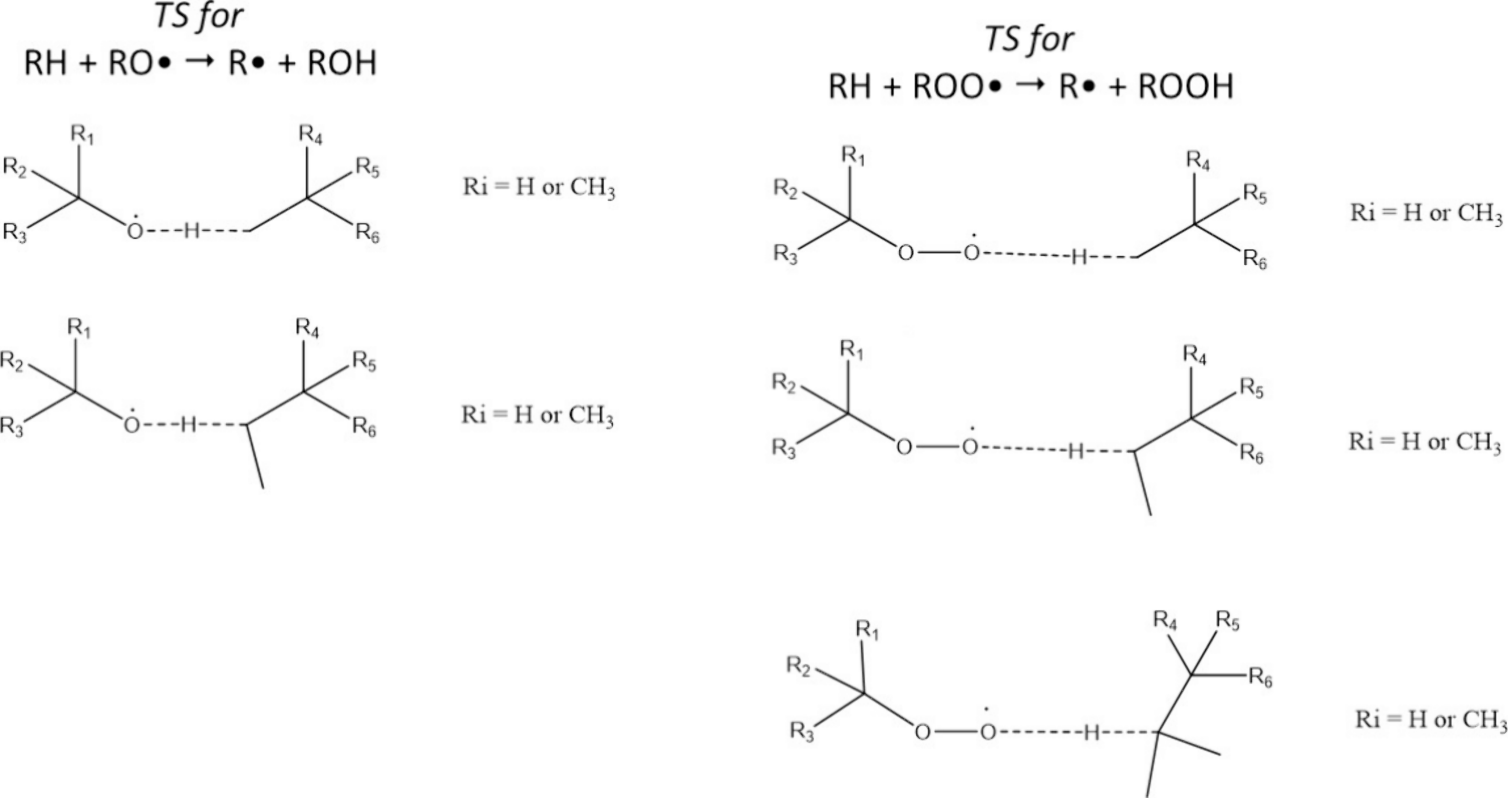
Representation of the TS generated for RH + RO· and RH + ROO· reactions with all combinations of R_i_ substituents (H or CH_3_) considered.

The development of a group contribution method for TSs is complex, since the development of a sufficiently large database to optimize the contribution of a given group is not straightforward. Here, we have used and adapted a recently published in-house code for the automatic calculation of kinetic data for combustion reactions.^77,78^ This code, written in Python, is designed to generate all possible combinations of methyl-substituted geometries based on the minimal structures, unsubstituted, reactant, TS, and product. These geometries are written in input files for quantum chemistry codes that are automatically run on high-performance computers. The code then retrieves the relevant information from the output files to calculate the rate constants. This capability has been used here to generate and optimize TSs for RH + RO and RH + ROO H-abstractions for alkanes. The methodology described above to compute the “TZVP-FINE” inputs for COSMOTherm was implemented in the code. The search for the lowest-energy conformer for each TS and each reactant was performed using relaxed scans (at the BP/TZVP level) of the single and transient (O--H--C in Figure 2) bonds in the geometries. More information on the identification and sorting of TS and reactants structures can be found in a previous study.^77^ The code was used to generate the COSMOTherm input files of the 80 TSs and their reactants, as summarized in Figure 5.

Moving down each column of structures in Figure 5, we see that all the H-abstractions of primary, secondary, and tertiary H atoms are described. For each TS represented, all the combinations of *R*_*i*_ substituents, which can either be a H atom or a CH_3_ group, lead to 16 structures.

#### Parameters , , and  from Critical Constants and Acentric Factors

3.2.3

As previously discussed, the parameters , , and  of any compound *i* can be estimated from group contribution methods. Their knowledge makes it possible to deduce the values of the critical temperatures () and the Soave α-function parameter (), which are used to describe the temperature dependence of the attractive parameter from the *PR* cubic EoS. In order to develop a group contribution method for these parameters, we need to develop a reference database of , , and , which will be used to fit the group contribution parameters. To generate this database, experimental values of , and  of closed-shell molecules were obtained from the *DIPPR* database and used to calculate , , and . It should be noted that  and  are calculated by eq 5, while the parameter  is calculated based on the Soave α-function at , as shown in eq 30.
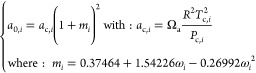
29Since , , and  are not experimentally available for free radicals and transition states, they were predicted using quantitative structure–property relationship (QSPR) models available in the *COSMOTherm* software. These empirical models correlate , , and  to parameters calculable by *COSMOTherm*, such as boiling temperatures and vapor pressures (see Appendix 2 for a more detailed discussion). In order to calculate these input quantities, it is sufficient to dispose of the COSMO file of the molecules of interest obtained through quantum-based calculations.

**Figure 6:**
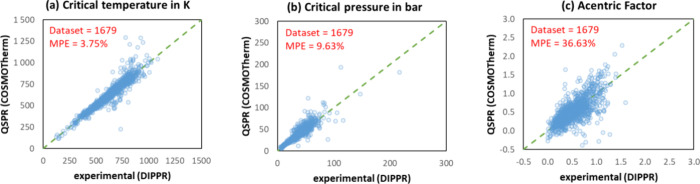
(a–c) Prediction of critical constants and acentric factors using the QSPR methods available in the COSMOTherm software.

Although these QSPR models provide reasonable predictions of , , and , they are not sufficiently accurate, especially for acentric factors, as pinpointed in Figure 6. The accuracy of the method was evaluated on a set of 1679 experimental values extracted from the DIPPR database related to stable molecules.

Despite the limited accuracy, this method is interesting because it only uses the COSMO file as input information, which can be done for any molecule of known geometry. In any event, it can be useful to provide initial guesses in a subsequent optimization procedure, as will be discussed below.

### 3.3 Parameterization of Functional Groups

Let us recall that the *tc*-PR/COSMO-RS EoS, introduced above, is a specific version of the PR EoS, embedding (i) the consistent Twu-91 α-function with a rigorous methodology for the determination of the α-function parameters of pure species, (ii) a volume translation parameter for pure species, and (iii) specific mixing rules for parameters  and , with  expressed as a function of the residual part of the excess Gibbs energy predicted by the COSMO-RS model. When group contribution methods are used to estimate the parameters of pure components, the *tc*-PR/COSMO-RS model no longer incorporates features (i) and ii but retains feature (iii). This is because, for the parametrization based on group contributions, we use the Soave α-function and approximate the volume translation parameter to zero for highly diluted solutes. In the next sections, we will present the methodology used to determine the group parameters used to estimate the pure compound parameters suitable for this version of the PR/COSMO-RS EoS.

#### 3.3.1 Multilinear Regression (GC-LR)

**Figure 7:**
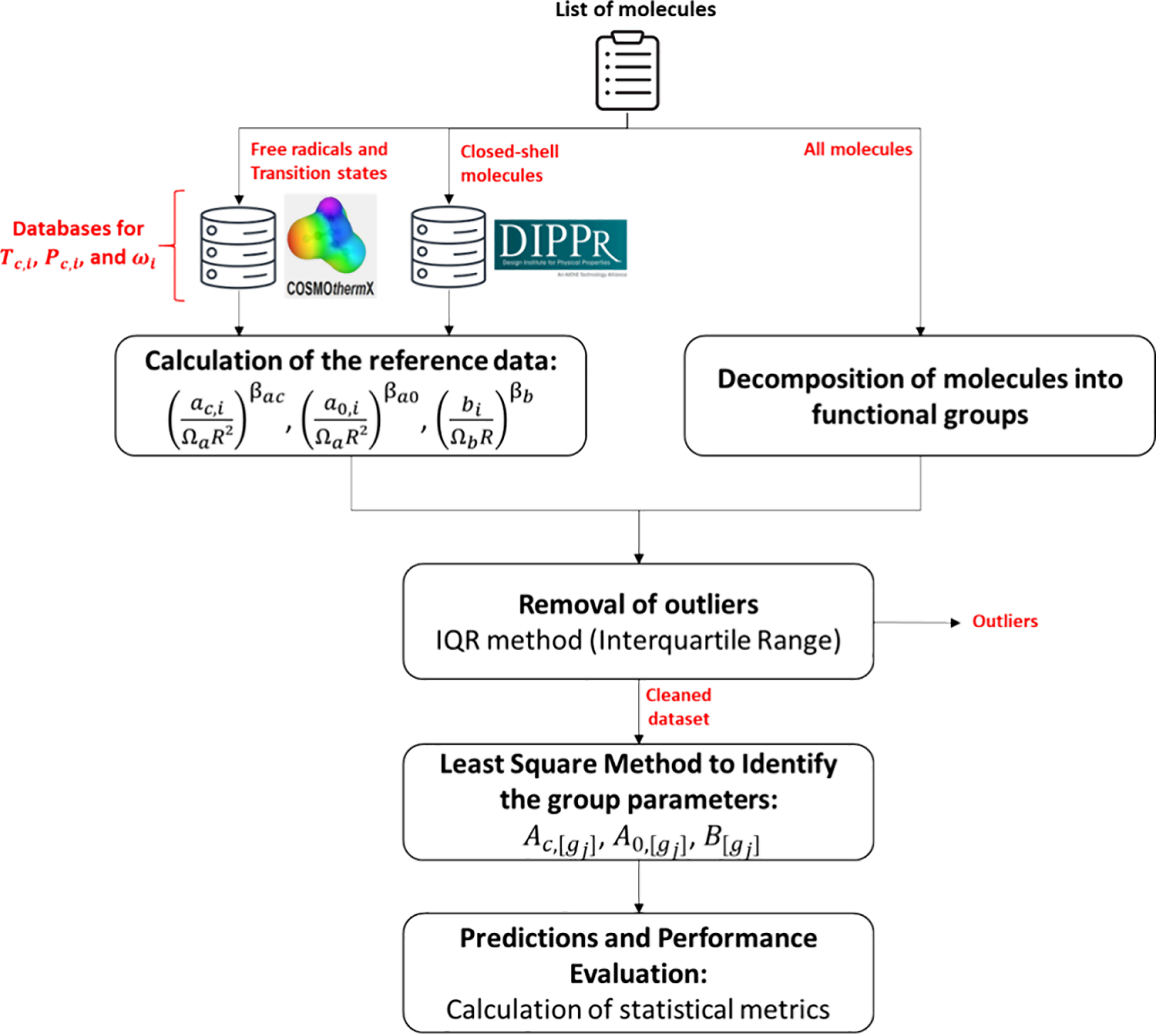
Workflow for fitting the contributions of functional groups through linear regression.

After establishing the reference database of , , and  parameters, the next step is to optimize the group contribution parameters. As the first-order group contribution method is a linear combination of the number of occurrences along with the contribution of each group, this optimization step can be performed by linear regression. This methodology is delineated in Figure 7 and will henceforth be termed as GC-LR, which stands for *group contribution-linear regression.*

As mentioned above, the Peng–Robinson EoS parameters , , and  were calculated first based on critical constants and acentric factors (experimental values for closed-shell molecules, and QSPR predictions for free radicals and transition states). Following this, in order to prevent numerical bias, the interquartile range (IQR) method was employed across the different chemical families in order to identify and remove outliers, such as the first molecule in a congeneric series, as in the case of methanol in the n-alcohol family. For the important molecules that were considered as outliers (such as methanol), a specific functional group composed of the entire molecule was introduced. Thereafter, a linear regression on the clean data set was performed to fit the group contribution parameters. The performance of the GC models was then evaluated using two key metrics: the mean percentage error (MPE) and the coefficient of determination (*R*^2^).

The methodology for estimating the group contributions for the generation of -profiles is essentially analogous to that presented in Figure 7 and was applied to the same molecules of the final training database. It is worth emphasizing that σ-profiles are discrete distributions involving 61 classes Δσ, and, as such, the aforementioned linear regression methodology was applied to each discrete value of σ, ranging from −3.0 to +3.0 with a step size of 0.1. This results in 61 linear regression models.

#### 3.3.2 Fine-Tuning of Group Contribution Parameters from Solvation Data (GC-NM)

Since the accuracy of QSPR methods is limited, a complementary optimization procedure was implemented, aimed at fitting the group contribution parameters of the , , and  properties of radicals and TSs to pseudoexperimental solvation Gibbs energy data. To this end, a generic database of solvation free energies was generated by using COSMOTherm. This database comprises data for 126 free radicals and 80 transition states in 73 different solvents, covering a temperature range from 298 to 398 K. The COSMOTherm calculations were performed using the same level of theory as that employed for the generation of the COSMO files (BP-TZVPD-FINE), using the BP-TZVPD-FINE-2016 parametrization (the same as the one used in our previous study). This data-driven approach was chosen based on COSMOTherm's capability to provide solvation data with a good accuracy level.^31^

The group contribution parameters for the , , and  properties were refined by further fitting them to the COSMOTherm data using a Nelder–Mead optimization algorithm (implemented in MATLAB). A good initial guess for the parameters was ensured by preadjusting them to the , , and  values generated by the QSPR models through multilinear regression (version GC-LR). This initialization was, therefore, the starting point for minimizing the objective function () defined in eq 31, which corresponds to the mean quadratic deviation between the COSMOTherm data and the corresponding data calculated from the *tc*-PR/COSMO-RS EoS.
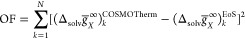
30

In the equation above,  is the value of the solvation free energy at infinite dilution of the solute molecule *X* calculated by the *COSMOTherm* software, and (Δ_solv_*g*_*X*_^∞^)_*k*_^EoS^ is the solvation free energy at infinite dilution of the same solute calculated with the *tc*-PR/COSMO-RS EoS, for the *k*-th of *N* data points.

The calculations of solvation data with the *tc*-PR/COSMO-RS model were carried out as follows:

- *Solvent molecules*: the parametrization of the pure compound inputs was done using experimental values of critical constants and acentric factors, optimized Twu-91 α-function parameters, and volume translation constants. Moreover, quantum-based -profiles were used. These parameters were taken from DIPPR, the publication by Piña-Martinez et al.,^40^ and the COSMOTherm database, respectively. Although solvation free energies in the liquid phase are often presumed to be independent of pressure, calculations employing an EoS require a pressure value as an input. Therefore, the calculations were conducted at a pressure equivalent to the vapor pressure of the liquid solvent at the specified temperature of each data point (thus ensuring that under such conditions, the EoS predicts a liquid phase).
- *Solute molecules*: all the EoS inputs (*T*_c_, *P*_c_, ω, and σ-profiles) were obtained by applying the group contribution methods outlined in Section 2.5. They were then used to estimate their solvation Gibbs energies in the pure solvents.
- The group contribution parameters for the , , and  properties of free radicals and transition states were the decision variables for the optimizations.

The version of the PR/COSMO-RS model relying on updated group contribution parameters for free radicals and transition states of the GC-based EoS is denoted “GC-NM”, abbreviation for g*roup contribution - Nelder Mead* optimization.

#### 3.3.3 Evaluating Model Performance for the Calculation of Solvation Data

Once all the group parameters were optimized, the performance of the PR/COSMO-RS model for predicting solvation energies was evaluated on three databases: one for closed-shell solutes, another for free radicals, and another for transition states. The database for closed-shell solutes was extracted from the CompSol experimental database.^38^ Only the data points that met the criteria below were selected:

- The critical temperature, critical pressure, volume translation parameter, Twu-91 α-function parameters, and quantum-based σ-profiles of the solvent molecule are known.
- The solute molecule could be decomposed into the functional groups available in this work. To this end, an automatic decomposition algorithm using as input the Simplified Molecular Input Line Entry System (SMILES) notation was implemented, based on the methodology implemented by Mülller.^79^

By applying these filters, around 15% of the points from the original CompSol database were disregarded, resulting in the database summarized in Table 7. It should be noted that, for this type of molecule, the fine-tuning step based on solvation data was not performed since the linear regression procedure had already been performed on accurate , , and  data extracted from the DIPPR experimental database. Therefore, only the “GC-LR” version was evaluated for these molecules. For C, H, and O free radicals and transition states, the same COSMOTherm databases used to fit the group parameters were considered for the analysis of deviations in the prediction of solvation energies. In this case, both the GC-LR and GC-NM versions were tested against the reference data generated by COSMOTherm, which is also outlined in Table 7. Using these databases, it was also possible to compare the solvation Gibbs energies of activation for each H-abstraction reaction calculated by the proposed approaches and by COSMOTherm.

**Table 7:** Different Solvation Free Energy Databases According to the Nature of the Solutes (Closed-Shell Molecules, Free Radicals, and Transition States)

	closed-shell molecules	free radicals	transition states
num. of solutes:	718	126	80
num. of solvents:	616	73	73
temperature range in K:	102–653	298–398	298–398
size of the data set:	60,559	45,990	29,195
source:	CompSol^38^	COSMOTherm	COSMOTherm

## 4 Results and Discussions

### 4.1 Obtention of Group Contribution Parameters through Linear Regression (GC-LR)

**Figure 8:**
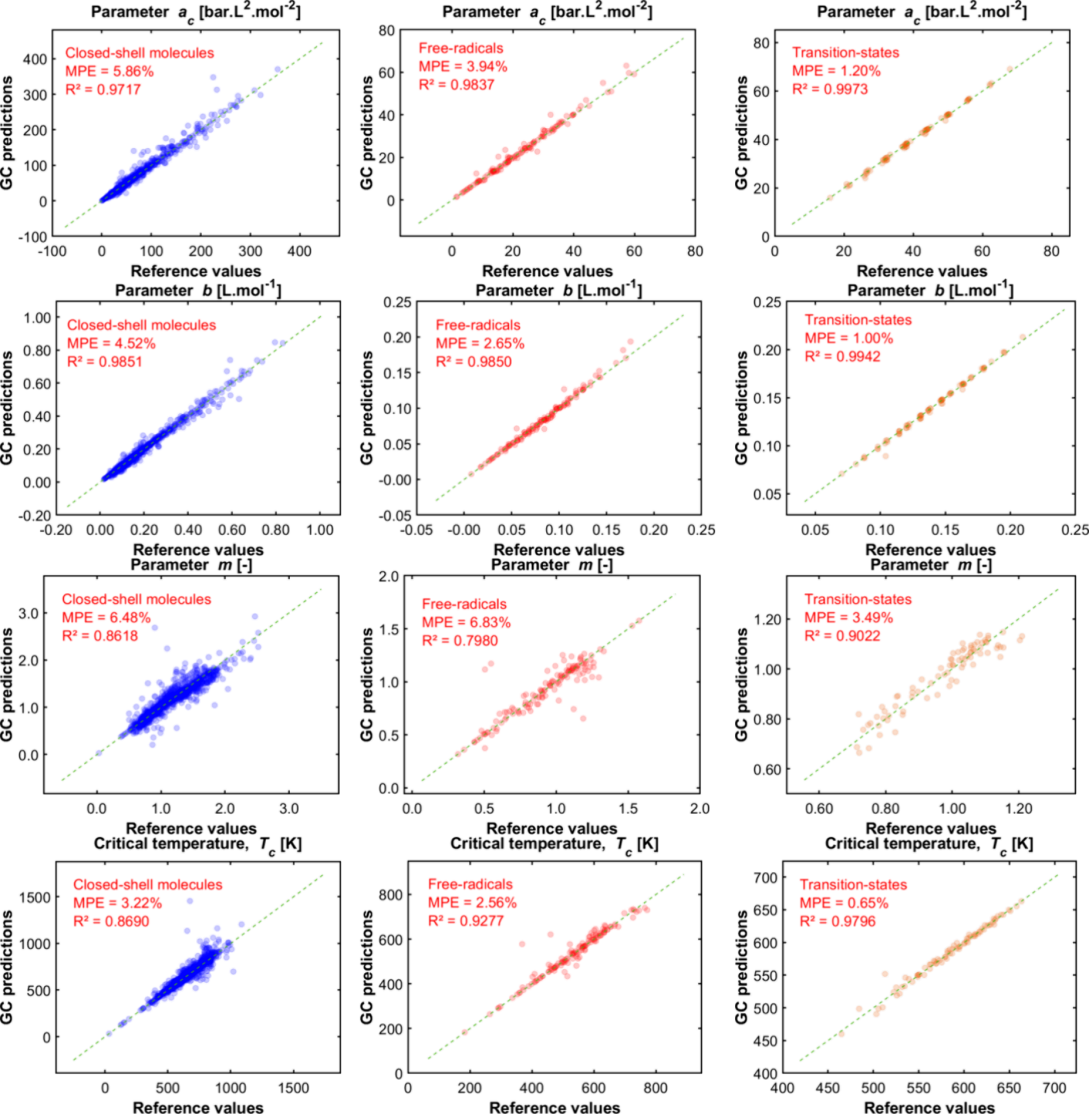
Parity plot between the reference data and the predictions by group contribution (GC) of the parameters of the *tc*-PR/COSMO-RS EoS (version “GC-LR”). MPE stands for “mean percentage error”, and *R*^2^ is the coefficient of determination.

The comparisons between the results obtained for the pure compound parameters (, , and ) for the *tc*-PR/COSMO-RS EoS via group contribution (the group contribution method used here is “GC-LR”) versus reference values are presented in Figure 8, which is divided into the different types of molecules (closed-shell molecules, free radicals, and transition states). It is recalled that the shape parameter  of the Soave α-function is deduced from the parameters  and , calculated from a group contribution method, and that  is deduced from  and that are also calculated from a group contribution method.

Since it is an updated version, the general conclusions are similar to those of our previous publication:^33^ the properties (*a*_c_, *m*, *b*, *T*_c_) of most organic families are predicted with a satisfactory degree of accuracy (MPE < 10%), especially those comprising C/H/O molecules. As highlighted in the past work, the prediction quality is lower for the parameter  than for the other parameters. It is recalled that  is directly related to the acentric factor, which is challenging to predict due to its complex link with the size, shape, and polarity of molecules.

Regardless of the parameter being evaluated, discrepancies were more pronounced in the case of polyfunctional compounds because, in these molecules, the environment around the functional groups (e.g., proximity of two polar groups, which can generate intramolecular hydrogen bonds) and their conformational configurations may have a significant impact on their properties, and these effects cannot be described by a first-order group contribution model. In this type of model, the groups are simply added together without accounting for any intramolecular interactions between them, either energetic or conformational.

**Figure 9:**
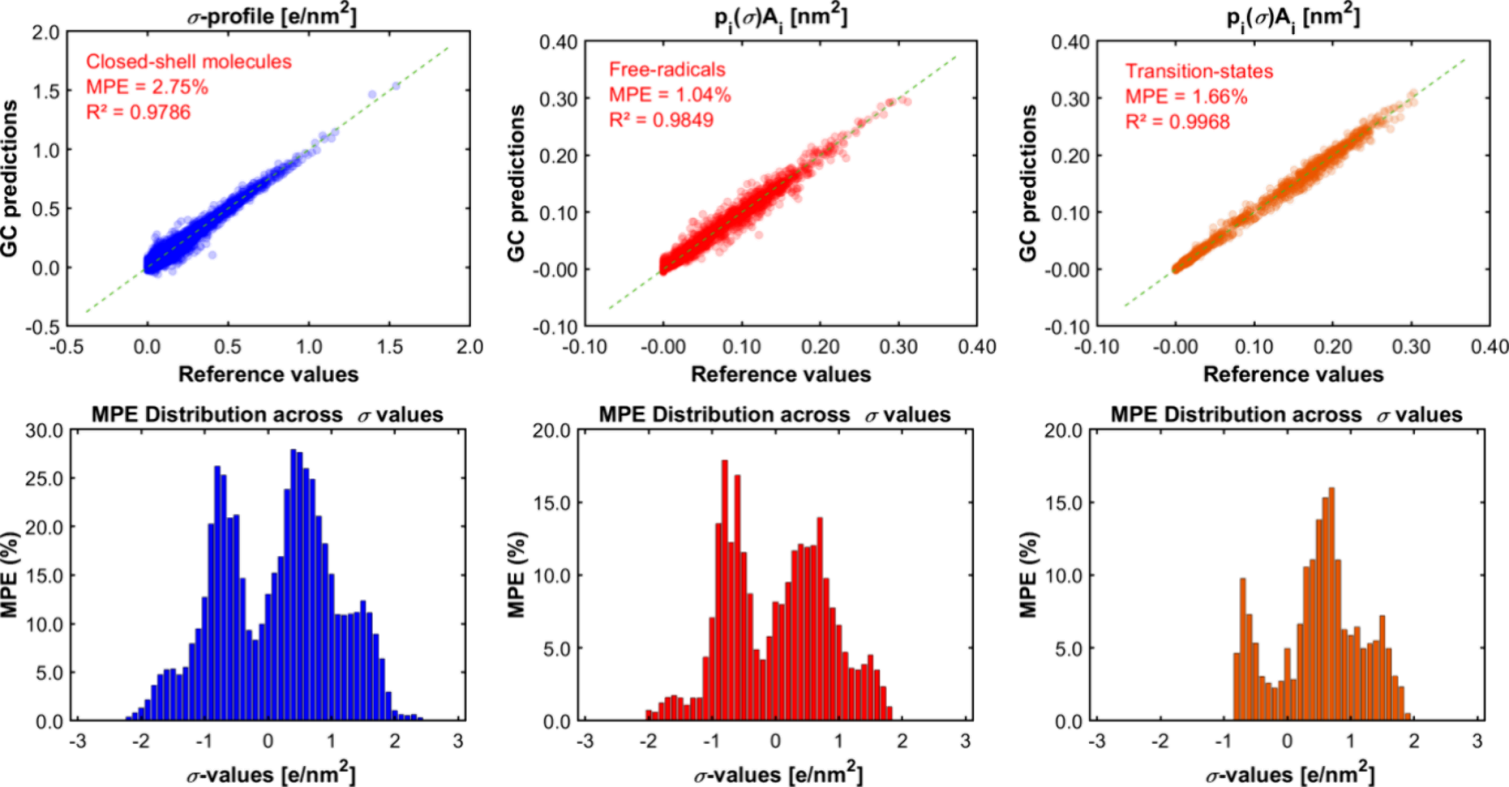
Parity plot between the reference data and the predictions by group contribution (GC) of the COSMO-RS σ-profiles and distribution of local deviations (in terms of MPEs) according to the value of the charge density (σ). In the parity plots, in particular, the MPEs (mean percentage errors) were determined as the absolute relative deviation in the area under the curve between the profile computed by using quantum calculations at the BP-TZVPD-FINE level and the profile derived from group contribution.

Similar conclusions can be drawn for the prediction of -profiles, whose metrics and plots are presented in Figure 9. In general terms, the GC-based model is able to reproduce the -profiles with higher deviations in the low-polarity region, for values between −1.0 and +1.0 e/nm^2^. A more detailed discussion about the accuracy of the group contribution method for predicting -profiles can be found elsewhere.^32,80,81^

For the particular case of free radicals and transition states, the results are compared with the QSPR methods available in *COSMOTherm* software. This partly explains the good performance of the GC methods, which is corroborated by the fact that only monofunctional C/H/O molecules are considered. In any event, these results are intermediate, since further optimization of the group contribution parameters was carried out, given that QSPR methods provided by the COSMOTherm software generally offer limited accuracy prediction, especially for acentric factors (and consequently the Soave α-function parameter). It should also be emphasized that this complementary optimization was only carried out for free radicals and transition state groups. For other groups, the parameters generated via linear regression were maintained, since they were fitted to real experimental data.

### 4.2 Estimation of Solvation Gibbs Energies of Closed-Shell Molecules

Turning now to the solvation Gibbs energies of closed-shell solutes at infinite dilution, our analysis of the results will once again be categorized according to the association behavior (by hydrogen bonding) of the binary mixtures, following the notation of the binary association codes (BAC), which summarizes the nature of the association established in a binary mixture.^82^ For the BAC notation, we first sort the compounds into four categories:

- NA = nonassociating, nonpolar (alkanes)
- HA = hydrogen-acceptor, polar but nonassociating (ketones, aldehydes, and ethers)
- HD = hydrogen-donor, polar but nonassociating (di- or trihalogenated compounds)
- SA = self-associating, polar, and associating (water, alcohols, and carboxylic acids)

Each notation is then combined to categorize the binary mixture association behavior, as shown in Table 8.

**Table 8:** Binary Association Code (BAC)

		solute			
		NA	HA	HD	SA
solvent	NA	NA-NA (BAC 1)			
	HA	HA-NA (BAC 2)	HA-HA (BAC 4)		
	HD	HD-NA (BAC 3)	HD-HA (BAC 6)	HD-HD (BAC 4)	
	SA	SA-NA (BAC 5)	SA-HA (BAC 8)	SA-HD (BAC 7)	SA-SA (BAC 9)

Mixtures with BAC values between 1 and 4 indicate that there is no association among the components. A BAC value of 5 corresponds to mixtures where only self-association occurs (association between molecules of the same species), as one of the components is a nonassociating molecule. A BAC value of 6 indicates mixtures in which cross-association occurs, with the components exhibiting no association in their pure states but forming hydrogen bonds when mixed. BAC values from 7 to 9 represent mixtures in which both cross- and self-association take place.

**Figure 10:**
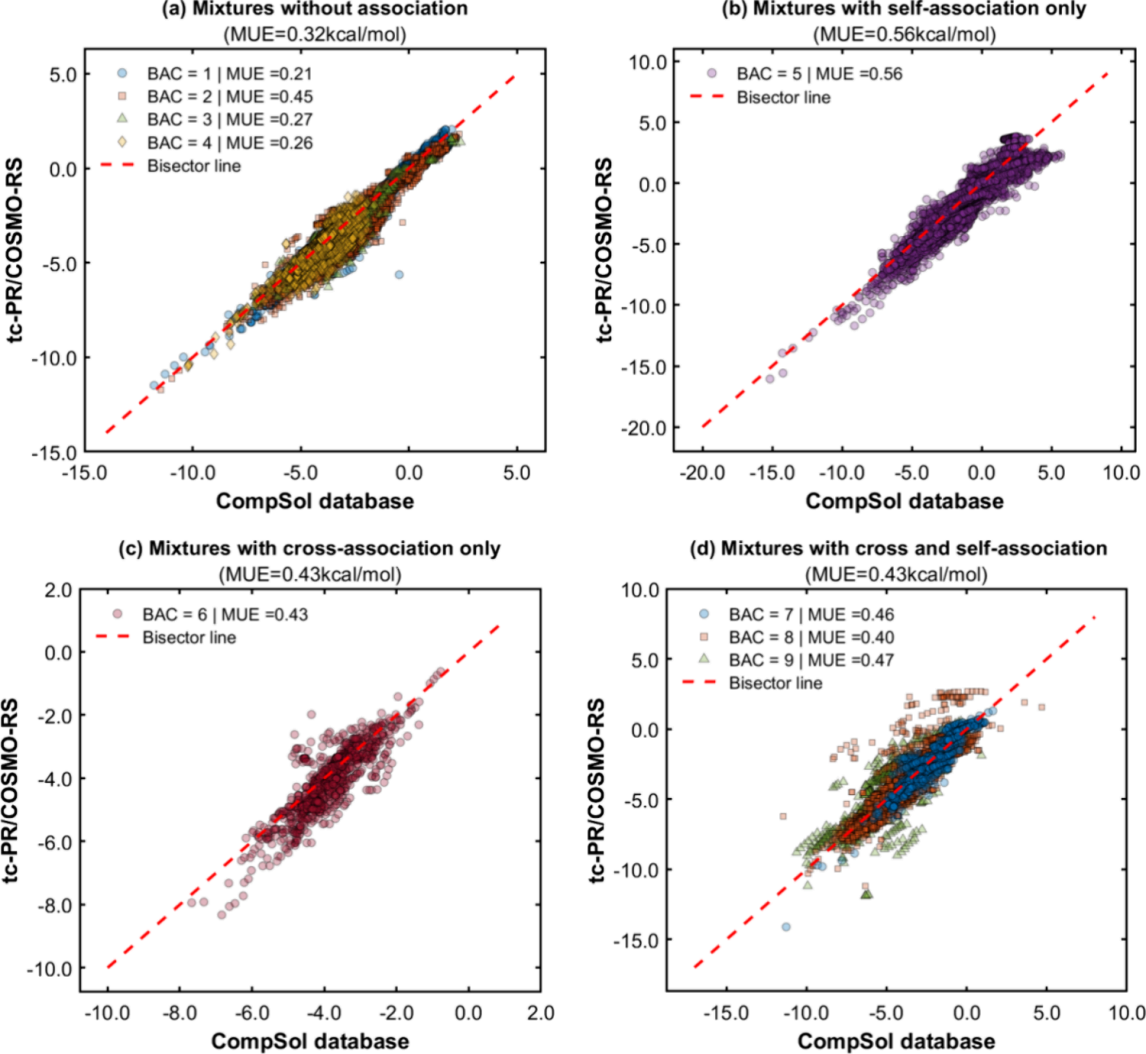
(a–d) Parity plots between experimental data from the CompSol database and calculated data obtained with the GC-based *tc*-PR/COSMO-RS EoS for the solvation Gibbs energy of closed-shell solutes infinitely diluted in liquid solvents. The pure compound inputs of the solute molecules were predicted by using the proposed group contribution methods.

Figure 10 provides parity plots comparing the experimental solvation free energies from the CompSol database with those calculated by using the GC-based *tc*-PR/COSMO-RS EoS, according to the classification of the binary association code (BAC). Although most of the solutes have known experimental critical constants, Twu-91 α-function parameters and quantum-based σ-profiles, it has been decided to systematically use the group contribution approach to obtain the pure compound inputs of the solute molecules for the *tc*-PR/COSMO-RS EoS in order to benchmark the fully predictive version of the *tc*-PR/COSMO-RS EoS. It should be noted that for close-shell molecules, only the GC-LR version of the group contribution methods is used, while the *GC-NM* version will be applied only to free radicals and transition states.

As expected, the most accurate metrics were obtained for mixtures with no association effects (BACs 1–4), with a mean absolute deviation of 0.32 kcal/mol. BAC 5 was the one that showed the worst correlation with experimental data (MUE = 0.56 kcal/mol) as it involves complex mixtures whose components have very different chemical natures, such as hydrocarbons with alcohols or carboxylic acids. Challenges were observed in the representation of solvation Gibbs energies of binary mixtures containing molecules capable of forming complex association schemes, such as some binary systems from BACs 7 to 9. This is mainly due to the inherent difficulties in representing association by hydrogen bonds with a predictive thermodynamic model. These challenges arise from the highly directional nature of this type of interaction, in addition to their sensitivity to temperature, varying strengths, and complex dynamics. Although the COSMO-RS model includes a generalized hydrogen bond interaction term, many of these localized effects are omitted or treated empirically, which reduces the accuracy of the model for representing complex cases.

**Figure 11:**
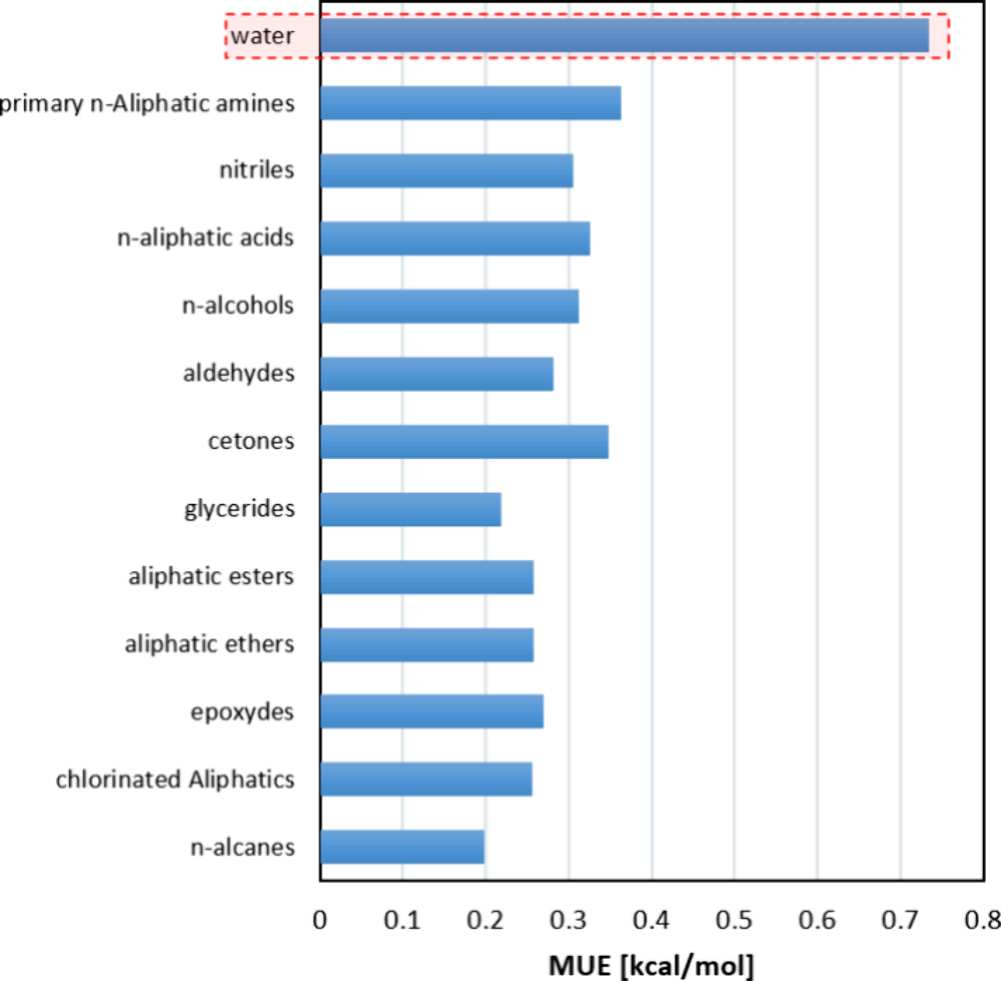
Average deviations obtained in organic solvents and water for the prediction of solvation energies with the GC-based version of *tc*-PR/COSMO-RS EoS.

In terms of complexity, water is the most common substance where hydrogen bonding is crucial, but its behavior is notoriously difficult to model. Each water molecule can form up to four hydrogen bonds, resulting in extensive and constantly shifting networks, with hydrogen bonds breaking and reforming. Some of the peculiar behavior of pure water—e.g., the density anomaly (water is less dense as a solid than as a liquid), high heat capacity, and surface tension—are partly explained by the complexity and strength of its hydrogen bonds. To summarize, representing pure water is not an easy task for any thermodynamic model, and its behavior in a mixture becomes even more challenging. This was indeed observed in our results regarding the hydration Gibbs energies (i.e., the solvation Gibbs energy in water). As shown in Figure 11, the average deviations tend to be higher when water is the solvent compared to other organic solvents.

The results for most organic solvents were satisfactory, with deviations close to 0.3 kcal/mol with respect to the CompSol database. However, the average deviations increased in systems involving strongly polar solvents, such as polyols, where deviations exceeded those observed in water, reaching 1 kcal/mol. Polyfunctional solvents containing various polar groups capable of performing hydrogen bonding also exhibited a poorer correlation with the experimental data. This discrepancy is likely due to their complex association behaviors, in addition to conformational effects, which are difficult to accurately model within the COSMO-RS framework. One way to improve the modeling of association and hydrogen bonding (and, at the same time, keeping the modeling simple and straightforward) is to introduce universal constants that are based on the nature of the interacting atoms in a hydrogen bond. For instance, the constant representing the interaction between two hydroxyl groups (OH) should be different from the constant representing the interaction between an OH group and a C=O carbonyl group. This concept is implemented in alternative versions of COSMO models, such as COSMO-SAC 2010^83^ and COSMO-SAC-dsp.^84^

Besides the challenges in modeling hydrogen bonds, it is important to note that the input information about the solutes was generated using group contribution methods, both for the parameters of the Peng–Robinson EoS (, , , and ) and for the σ-profiles () used in the calculation of the residual activity coefficients within the mixing rules. To identify the primary source of error that arises from the different group contribution methods, Table 9 presents four distinct scenarios:

- Scenario 1: Both the parameters of the PR-EoS and the σ-profiles are used in their original forms, obtained from experimental data (, , and  are calculated from experimental values of , , and , respectively) and quantum calculations (σ-profiles). This is the most accurate scenario.
- Scenario 2: The parameters of the PR-EoS are kept as original (calculated with experimental data), while the σ-profiles are calculated using group contribution.
- Scenario 3: The parameters of the PR-EoS are calculated using group contribution methods, while the σ-profiles are based on their original form (obtained through quantum calculations).
- Scenario 4: Both the PR-EoS parameters and the σ-profiles are calculated using group contribution (same results as Figure 10). This is the least accurate scenario.

**Table 9:** Solvation Free Energy Results (in Terms of Mean Unsigned Error in kcal/mol) Are Categorized by the Type of Input Used for Describing the Solute Molecules and the Binary Association Code (BAC)^a^

		scenario 1	scenario 2	scenario 3	scenario 4
BAC	number of data	*PR*-exp *p*(σ)-QM	*PR*-exp *p*(σ)-GC	*PR*-GC *p*(σ)-QM	*PR*-GC *p*(σ)-GC
1	12,241	0.12	0.13	0.20	0.21
2	13,511	0.28	0.33	0.39	0.45
3	1252	0.18	0.20	0.24	0.27
4	3077	0.16	0.17	0.25	0.26
5	14,063	0.37	0.50	0.44	0.56
6	1005	0.41	0.40	0.41	0.43
7	1173	0.44	0.46	0.44	0.46
8	8190	0.36	0.38	0.39	0.40
9	6047	0.36	0.40	0.43	0.47
all	60,559	0.28	0.34	0.36	0.41

a ‘PR-EoS’ refers to inputs derived for the Peng–Robinson equation of state (with ‘exp’ denoting the use of experimental data and ‘GC’ indicating group contribution methods), while ‘*p*(σ)’ represents the mode used for generating σ-profiles (‘QM’ for quantum mechanical calculations).

For each scenario, the PR-EoS parameters and the σ-profiles of solvent molecules were obtained from experimental data and quantum calculations.

The first scenario is clearly the most accurate, as it does not rely on any group contribution input. For closed-shell solutes, this scenario is preferred for kinetic simulations because the required inputs for these solutes are readily available from the existing databases. The other scenarios are therefore designed to assist when input information for a particular solute is unavailable. In such cases, the results indicate that the error associated with calculating PR-EoS parameters using group contribution methods is slightly larger than the error obtained with σ-profiles generated from group contribution methods. However, it is important to keep in mind that while the proposed first-order group contribution models introduce additional inaccuracies to the model, they fully bypass the need for experimental data and quantum-based inputs, thereby making the equation of state fully predictive, more flexible, and suitable for high-throughput applications. Moreover, the use of simplified group contribution methods is easier to implement and automate within kinetic generators (the decomposition of molecules into first-order group contributions is simpler to implement than higher-order group contribution schemes).

### 4.3 Calculation of Solvation Gibbs Energies of Radicals and Transition States

The analysis of the solvation Gibbs energies of closed-shell solutes provided insights into the strengths and limitations of the group contribution version of the *tc*-PR/COSMO-RS EoS. We will now extend this discussion to the solvation of free radicals and transition states. For the following analyses, we will continue using experimental inputs (critical temperature, critical pressure, and parameters of the Twu-91 α-function) and quantum-calculated σ-profiles for the solvent molecules. Likewise, the stable reactants in the solvation Gibbs energy of activation analysis will follow the same approach, while group contribution methods (GC-LN and GC-NM) will be applied to free radicals and transition states.

#### 4.3.1 Using GC-LR Parametrization (Multilinear Regression on QSPR Data)

**Figure 12:**
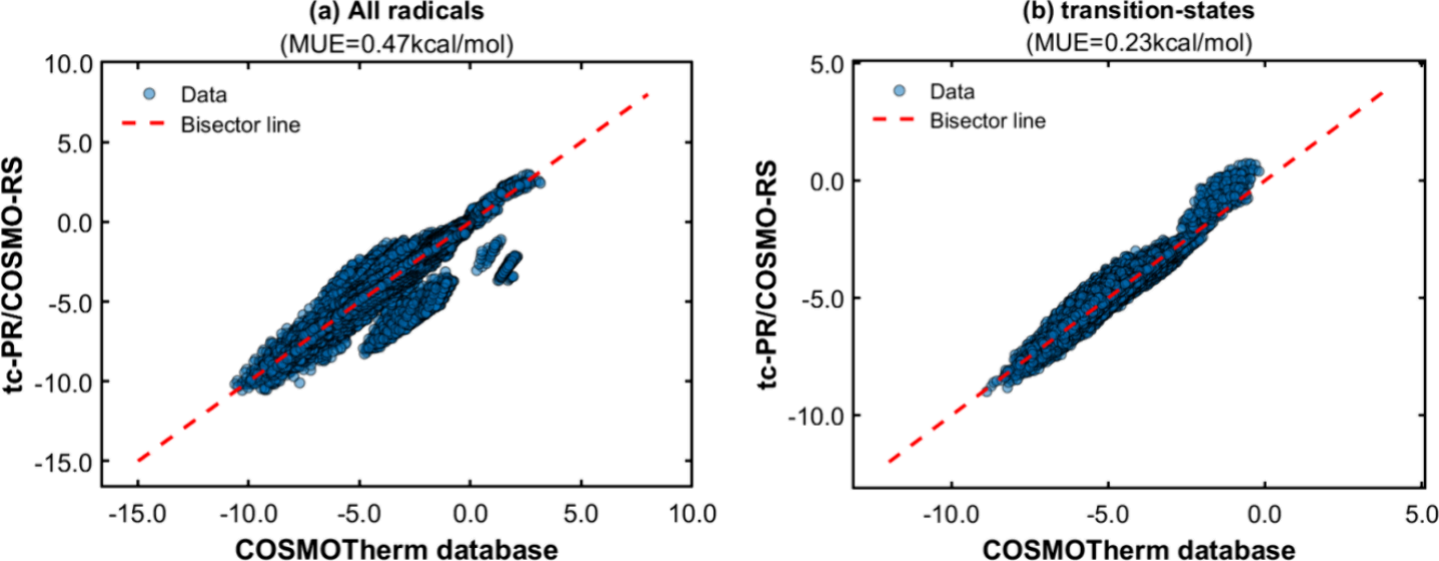
(a, b) Parity plots between pseudoexperimental data for the solvation Gibbs energy of C/H/O free radicals and H-abstraction transition states infinitely diluted in liquid solvents generated with COSMOTherm, in comparison to calculated data obtained with the GC-based (GC-LR) *tc*-PR/COSMO-RS equation of state.

In Figure 12, we first present the intermediate results obtained for the solvation Gibbs energies of 126 free radicals and 80 transition states in 73 different solvents by using the GC-LR version of the PR/COSMO-RS model. The contribution parameters of these transient molecules were determined from multilinear regression based on , , and  values generated via QSPR models, along with quantum-based σ-profiles. The reader should be aware that the analysis of the results obtained from the GC-based *tc*-PR/COSMO-RS EoS is being compared to those obtained from another model (the COSMO-RS implementation of COSMOTherm), rather than to real experimental data for the simple reason that it is not possible to find experimental data about mixtures containing free radicals or transition states.

**Figure 13:**
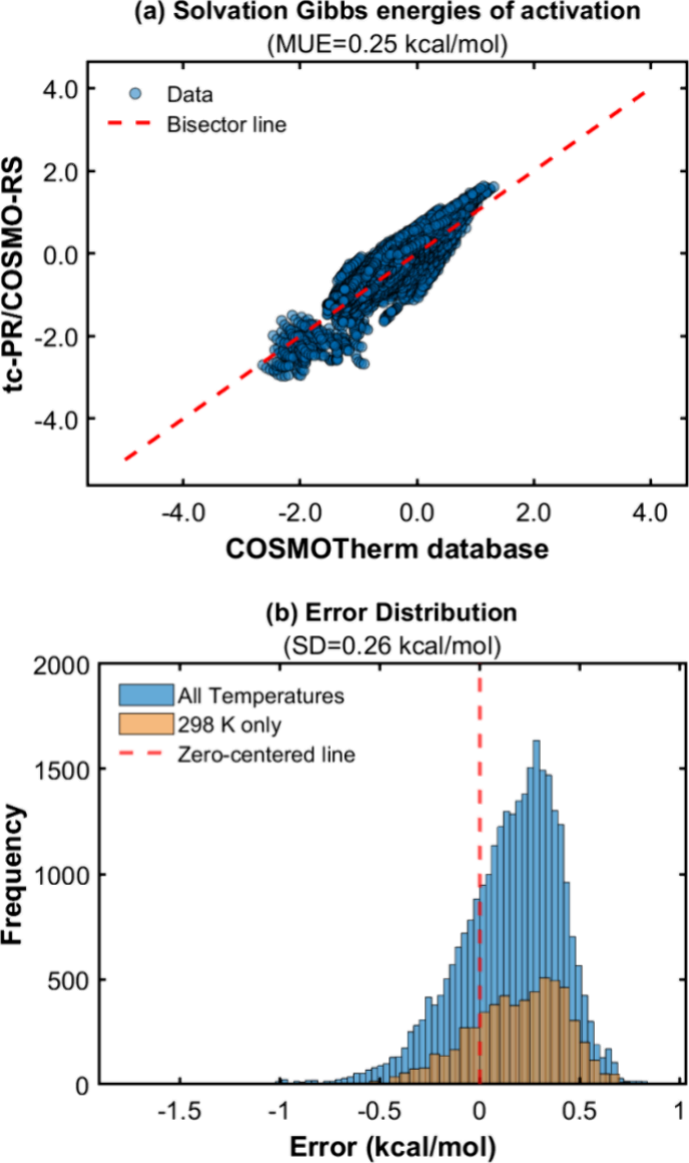
(a) Parity plot between pseudoexperimental data for the solvation Gibbs energies of activation of H-abstraction reactions in liquid solvents generated with COSMOTherm, in comparison to calculated data obtained with the GC-based (GC-LR) *tc*-PR/COSMO-RS EoS. (b) Error distribution for the solvation Gibbs energies of activation. The error is the difference between the data calculated by the equation of state and those calculated by the COSMOTherm software.

In general, the GC-LR version can provide reasonable estimates of the group contribution parameters for all transition states and for most free radicals considered in this study. Consequently, the mean absolute deviations in the solvation Gibbs energies calculated by the *tc*-PR/COSMO-RS model were found to be below 0.5 kcal/mol, with a noteworthy degree of precision observed for transition states. Overall, most free radicals were also reasonably well-represented, although larger deviations were observed for acetylenic radicals (which are the points outside the diagonal on the parity curve in Figure 12a) and some oxygen-centered radicals. Nonetheless, an accumulation of errors was observed regarding the calculation of solvation Gibbs energies of activation, as illustrated in Figure 13. This quantity turns to be very sensitive to the accuracy of the solvation Gibbs energy of the transition states and reactants involved. As a result, systematic errors were observed in certain reactions, leading to an error distribution that is skewed and not centered around zero, as shown in Figure 13b.

In view of the systematic deviations obtained for the solvation Gibbs energy of activation, an additional optimization step was performed (as described in Section 3.3.2) to enhance the accuracy of the group contribution models, with the view of better correlating the solvation data generated by the equation of state and by the COSMOTherm software. This updated version is termed GC-NM, and the results associated with this version are presented in the next section.

#### 4.3.2 Using GC-NM Parametrization (Nelder–Mead Optimization on Solvation Data)

**Figure 14:**
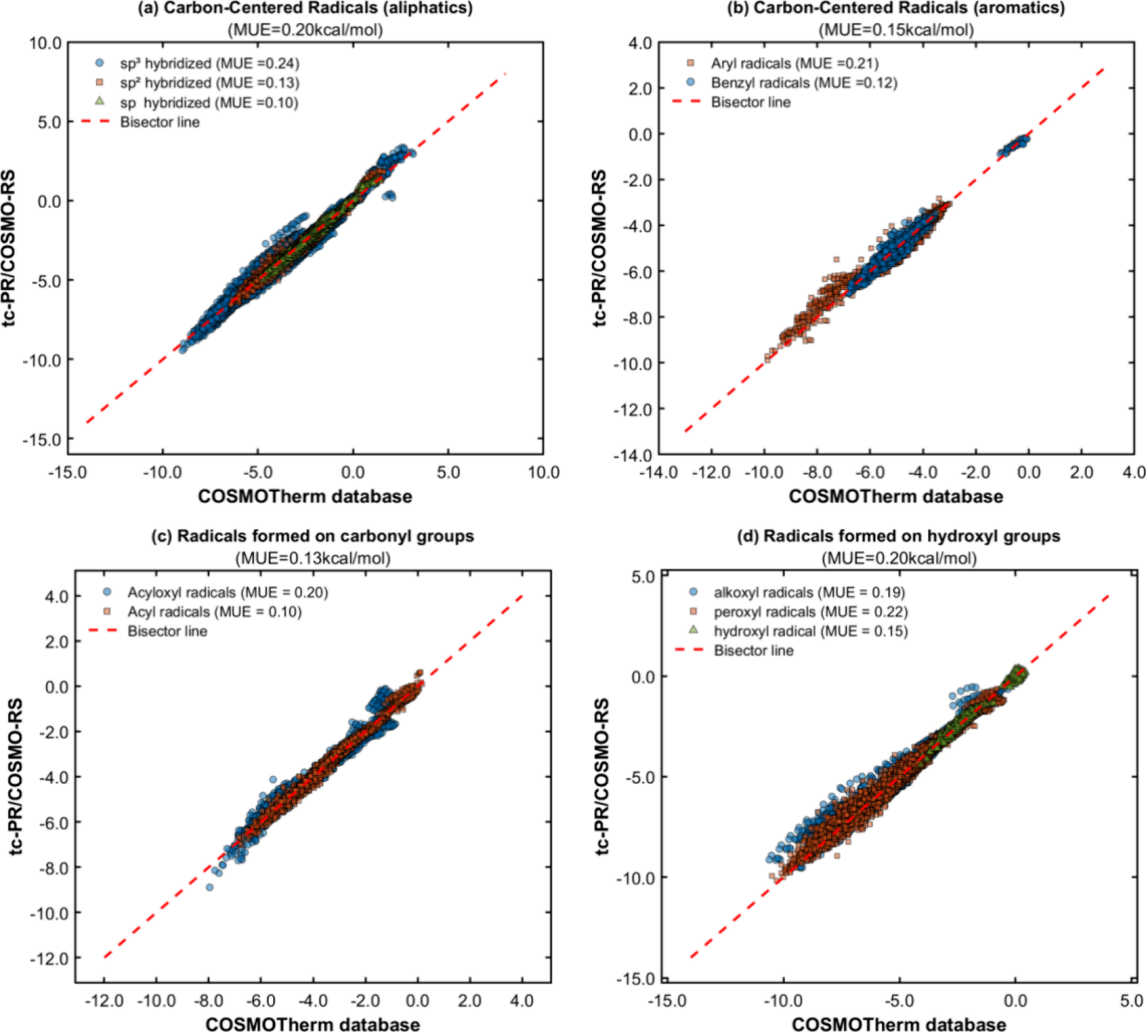
(a–d) Parity plots between pseudoexperimental data for the solvation Gibbs energy of C/H/O free radicals and transition states infinitely diluted in liquid solvents, in comparison to calculated data obtained with the GC-based (GC-NM) *tc*-PR/COSMO-RS equation of state.

Figure 14 summarizes the outcomes of the solvation Gibbs energy calculations using the *GC-NM* version of the group contribution methods to estimate the pure compound parameters (, , , and σ-profile) of the COSMO-based EoS. In order to provide a more detailed overview of the calculations, the results are classified according to the type of radical described in the methodology section.

**Figure 15:**
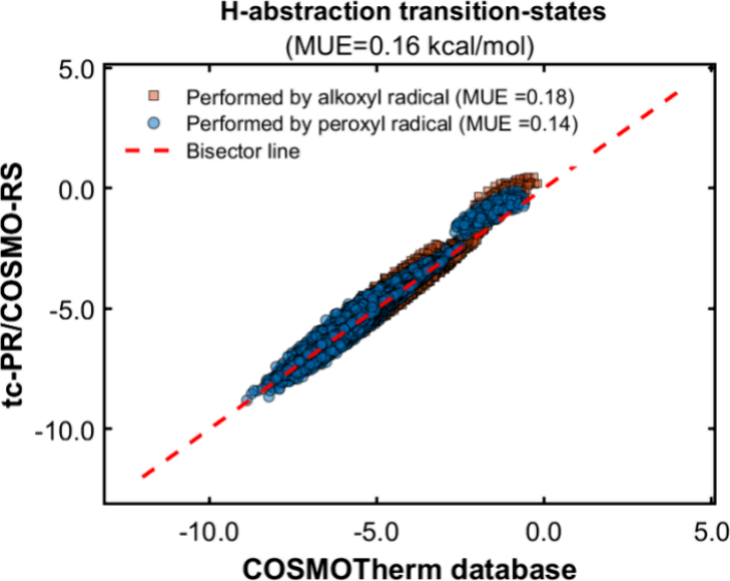
Parity plot between pseudoexperimental data for the solvation free energy of H-abstraction transition states infinitely diluted in liquid solvents, in comparison to calculated data obtained with the GC-based (GC-NM) *tc*-PR/COSMO-RS equation of state.

Figure 14 demonstrates an accurate reproduction of the data generated by *COSMOTherm*, with average deviations of less than 0.25 kcal/mol for all types of free radicals. For transition states, the solvation results obtained with the EoS are still in good agreement with those generated by COSMOTherm, with a mean absolute deviation of 0.16 kcal/mol observed across all of the H-abstraction reactions considered (see Figure 15).

**Figure 16:**
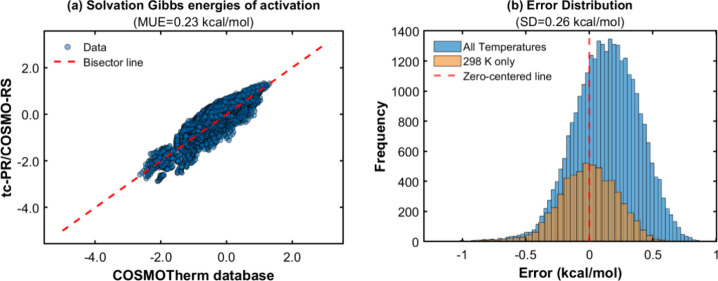
(a) Parity plot between pseudoexperimental data for the solvation Gibbs energies of activation of H-abstraction reactions in liquid solvents generated with COSMOTherm, in comparison to calculated data obtained with the GC-based (GC-NM) *tc*-PR/COSMO-RS equation of state. (b) Error distribution for the solvation Gibbs energies of activation. The error is the difference between the data calculated by the equation of state and the COSMOTherm software.

Figure 16 presents the results for the final variable of interest: the solvation Gibbs energy of activation, which depends on the solvation Gibbs energy of both the transition states and the reactants. For this solvation quantity, the average deviation obtained was 0.23 kcal/mol for the entire data set. Although this value decreased only by 0.02 kcal/mol compared to the previous case, the error distribution is better centered around zero, especially at 298 K.

**Figure 17:**
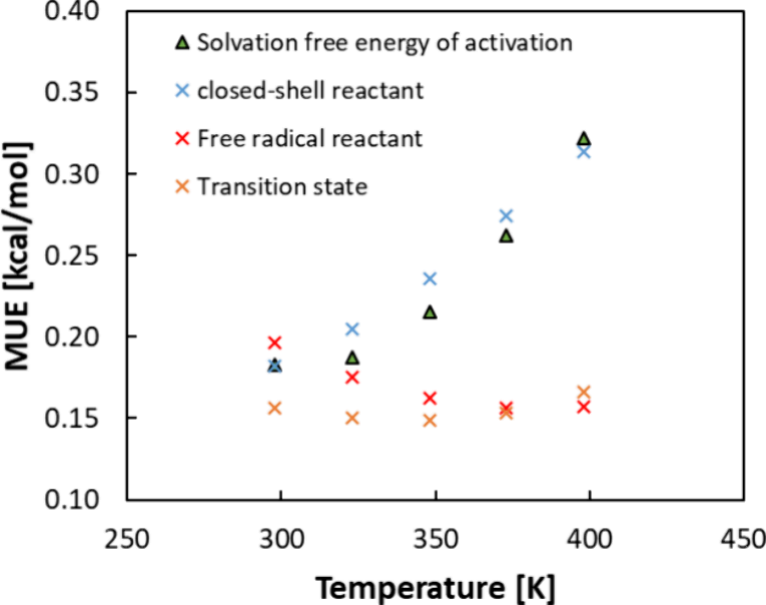
Mean unsigned error (MUE) between the predictions of solvation free energies with the GC-based (GC-NM) *tc*-PR/COSMO-RS EoS and COSMOTherm regarding the reactants and transition states of H-abstraction reactions. The MUEs are presented as functions of temperature.

Although the solvation Gibbs energies of activation calculated using the *tc*-PR/COSMO-RS EoS and COSMOTherm were in close agreement at 298 K, with an average absolute difference of only 0.18 kcal/mol, deviations were found to increase at higher temperatures, as illustrated in Figure 17. This increase is mainly associated with the calculation of the solvation Gibbs energies involving closed-shell reactants (the one that loses a H atom), which, unlike radicals and transition states, were not parametrized with group contributions fitted to data generated with COSMOTherm. For these transient molecules, a good agreement with the COSMOTherm results was observed for the entire temperature range considered.

**Figure 18:**
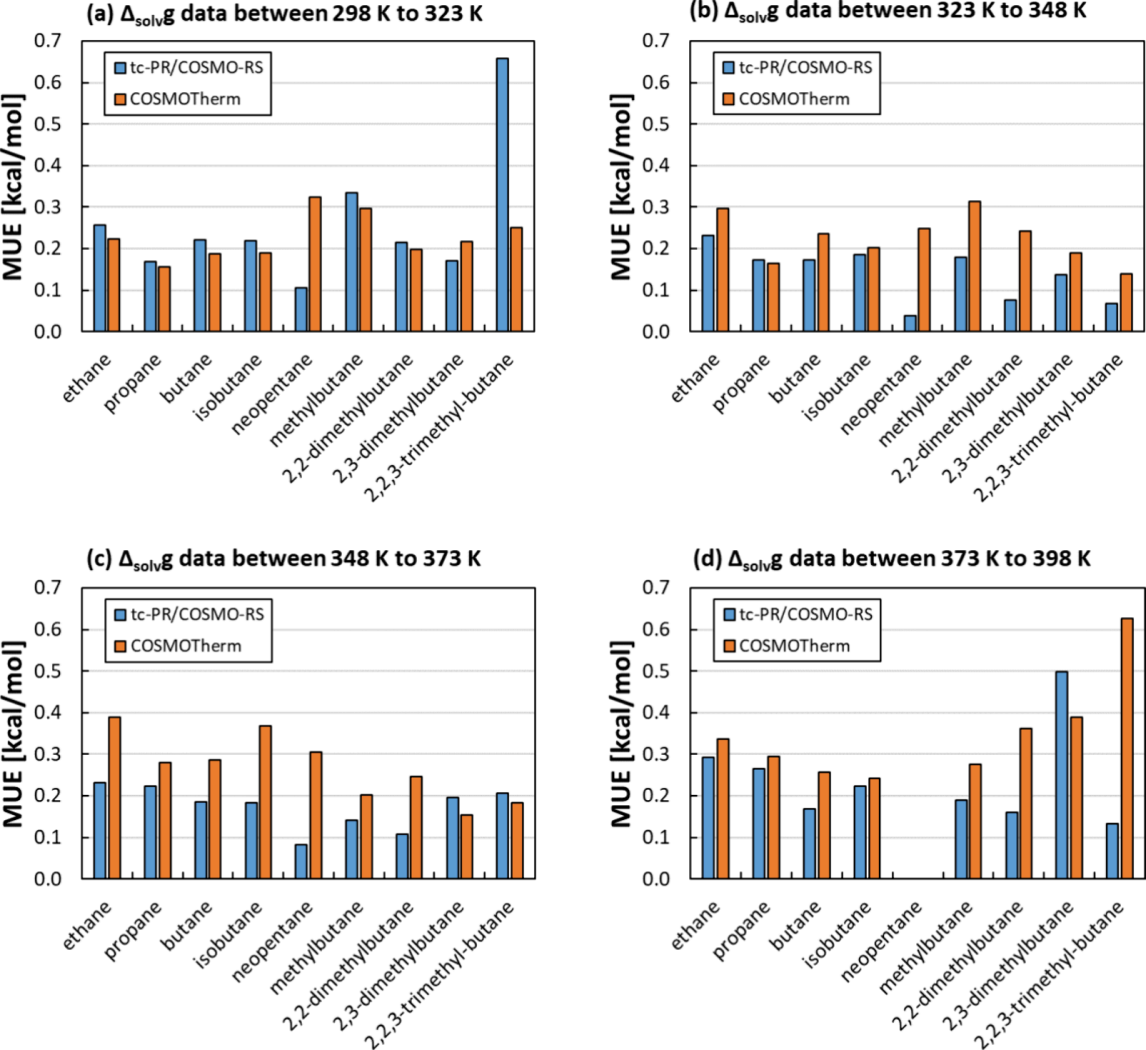
(a–d) Mean unsigned error (MUE) between solvation free energy predictions obtained using either the GC-based (GC-NM) *tc*-PR/COSMO-RS EoS or COSMOTherm software compared to experimental data from the CompSol database. The analysis includes reactants from H-abstraction reactions in various solvents and across different temperature ranges.

In the case of closed-shell reactants, the outcomes produced by COSMOTherm and the EoS can be contrasted with the experimental data from the CompSol database, thereby providing an overview of their performance across different temperature levels. Figure 18 demonstrates this by showing the mean unsigned errors for each closed-shell reactant involved in the H-abstraction reactions considered in this work, within different temperature ranges. These temperature intervals were selected to be consistent with those used to calculate the solvation Gibbs energies of activation (between 298 and 398 K).

**Figure 19:**
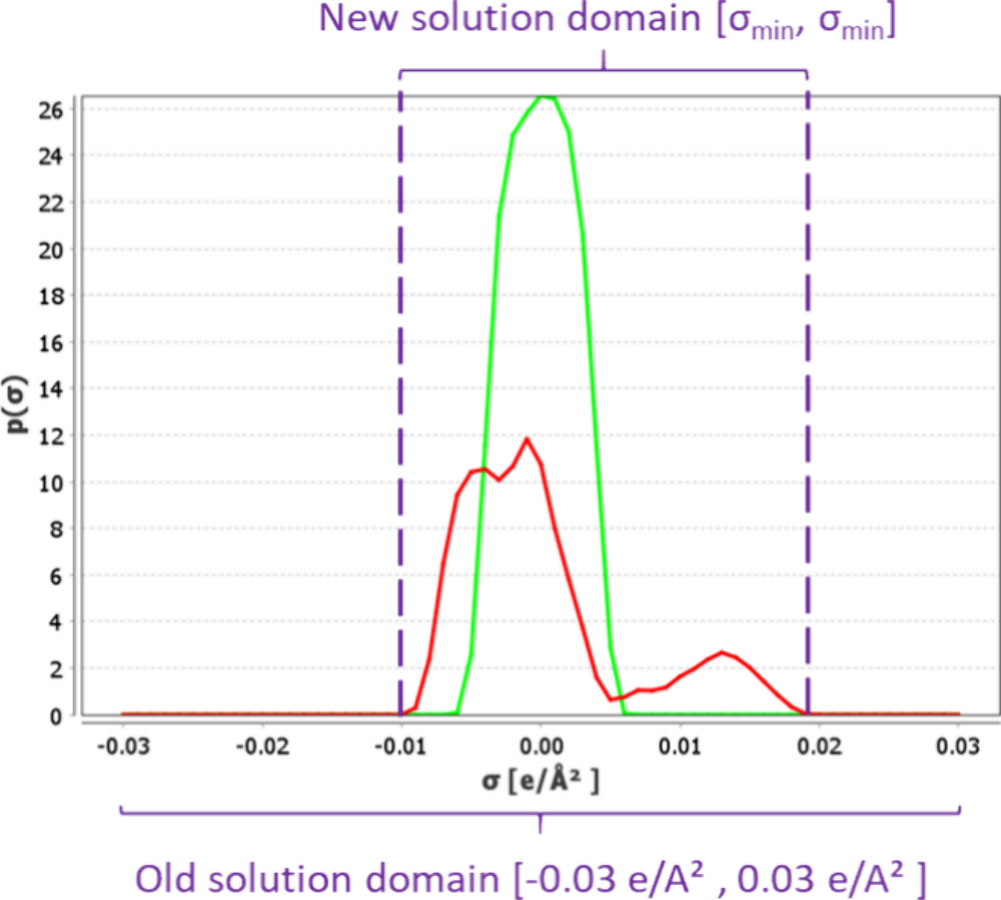
Reducing the solution domain for σ-potentials.

Figure 18 illustrates that, in most cases, the *tc*-PR/COSMO-RS model outperforms COSMOTherm, displaying lower mean unsigned errors (MUEs) for almost all of the data above 298 K. This suggests that the EoS approach is more precise in capturing the temperature difference of the solvation Gibbs energy of solute molecules under diverse temperature conditions. However, in order to better explore the temperature dependence of the solvation Gibbs energies, especially in the case of transient molecules (for which solvation energy data are not available), it is recommended to compare the predictions of kinetic constants in the liquid phase with experimental data available in the literature. For this purpose, in future work, we will calculate liquid-phase reaction rates by applying the solvation Gibbs energy of activation corrections to gas-phase reaction rates and subsequently compare these results with published reaction rate constants in liquids. This will allow for a more detailed assessment of the model accuracy and applicability across various conditions.

## 5 Conclusions

In this work, we implemented a predictive equation of state (called the *tc*-PR/COSMO-RS EoS) to calculate solvation free energies for a variety of closed-shell molecules, free radicals, and transition states at infinite dilution. The predictive power of the model comes from its reliance on pure compound inputs derived from group contribution methods, making it a robust tool for a wide range of solute molecules. A significant advance in this version of the *tc*-PR/COSMO-RS model is the inclusion of functional groups designed to represent transition states involved in hydrogen abstraction reactions. This enhancement has resulted in accurate predictions of solvation free energies of activation, with an average deviation close to 0.2 kcal/mol, especially for data close to 298 K. Several avenues for future work have been identified. These future directions hold great potential for further refining and expanding the capabilities of the proposed approach, which can be of great value for the design of complex chemical reactions. Among the main topics for future research, we can highlight:

- Expanding the database of transition states to include a wider variety of reactions, such as β-scissions reactions. This will enhance the model applicability.
- Improving the COSMO-RS model to lead more accurate results. One potential enhancement is to refine the modeling of hydrogen bonds, similar to the approach used in the COSMO-SAC 2010 model, where universal hydrogen bond constants are tailored to the specific nature of the atoms involved (O, N, or F). A benchmarking of different temperature functions to describe the universal constants could also be a promising improvement. Moreover, the incorporation of a dispersive term into the contact energy of the COSMO-RS model could better capture the subtleties of molecular interactions, especially for systems involving nonpolar molecules.
- Exploring the use of artificial intelligence methods for inputting solute molecules could be a significant advance. In most cases, a machine learning approach improves accuracy by overcoming some limitations inherent to group contribution models, such as their insensitivity to molecular conformations, and their restricted range of application to molecules that can be decomposed into the available functional groups.

## Data Availability

The “supporting information” file contains the lists of functional groups considered in this study concerning closed-shell molecules, C/H/O free radicals and transition states of hydrogen abstraction reactions. Moreover, the experimental and pseudoexperimental data used in this study, as well as the parameters of the group contribution methods developed here, are publicly available in the two files provided as Supporting Information (in XLSX format). The file “GC_parameters.xlsx” reports (i) the group contribution parameters for calculating the parameters *a*_c_, *b*, *a*_0_ of the Peng-Robinson equation of state, (ii) the group contribution parameters used for predicting the σ profiles. The complete σ profile is a histogram plotted for σ in the range −3 to +3 e/nm^2^ with a step of 0.1 e/nm^2^; therefore, the profile consists of 61 ranges of σ values and for each of them, one provides a set of group contribution parameters. The file “Gsolv_data.xlsx” reports: The solvation Gibbs energy data for infinitely diluted stable solutes in pure solvents: (i) extracted from the CompSol database and (ii) calculated from the PR/COSMO-RS EoS, the solvation Gibbs energy data for infinitely diluted C/H/O free radicals in pure solvents: (i) obtained from the COSMOTherm software and (ii) calculated from the PR/COSMO-RS EoS, the solvation Gibbs energy data for infinitely diluted H-abstraction transition states in pure solvents: (i) obtained from the COSMOTherm software and (ii) calculated from the PR/COSMO-RS EoS. The PR/COSMO-RS EoS calculations were implemented using in-house codes developed in MATLAB. These codes are publicly available on GitHub at this link https://github.com/FC-Paes/Peng_Robinson-COSMO_RS.git. The repository includes examples demonstrating the model’s implementation for pure liquid solvents and can be readily extended to handle mixtures.

## Appendix 1. Solving the σ-potential equation

In this section, we will briefly discuss how to solve the equation used for calculating σ-potentials. Before doing this, it is important to reduce the solution domain to enhance computational efficiency. This can be achieved by considering only  values that are greater than zero, as shown in Figure 19.

Subsequently, we proceed to solve the σ-potential with eq 15, which turns to be an implicit equation that requires an iterative solving procedure. The most efficient approach is to reformulate this equation into a vectorized form and solve it using matrix operations. For this purpose, we define a vector *Y* and a matrix *u* based on remaining values of charge density (σ), as follows:

31

32

Thus, eq 15 can be rewritten in a more simplified way:

33

which can easily be converted into a matrix form with unknown *Y*, as shown in eq 34:

34

A simple, yet effective way to solve eq 34 is by using a successive substitution method, such as the one presented below:

1. Initialization of the vector *Y* (such as setting all values to 1).
2. Recurrence procedure:
  35
  36
3. The recurrence procedure is repeated until: max{ |*Y*^new^ – *Y*^old^|} < Tolerance

Another option would be to solve eq 34 using a Newtonian-based approach. For this, a quasi-Newtonian method is preferable, to avoid inversion of the Jacobian matrix, which can be time-consuming. Since Newtonian methods are sensitive to initializations, a few iterations of the successive substitution method can be employed to provide a suitable initialization.

## Appendix 2. Calculating Critical Constants with COSMOTherm

Starting with the 2019 version of COSMOTherm, it is possible to use QSPR methods to estimate critical constants and acentric factors. First, the critical temperature () and critical molar volume () are calculated based on selected COSMOTherm outputs, including the normal boiling temperature (), the dielectric energy scaled by the compound surface (), refractive index (*n*_*i*_), and cavity molecular volume (). The relationships established between , , and the outputs of COSMOTherm are shown in eqs 37 and 38.

37

38

where the parameters  and  are generic constants. The values of  and  are then used to estimate the critical pressure , according to eq 39. Again, the constants *c* are generic to the model.

39

Finally, the acentric factor () is calculated from its definition, given by eq 40.

40

where  is the vapor pressure of component *i* when its reduced temperature () is equal to 0.7. It is recalled that the reduced temperature is the quotient between the actual temperature  and the critical temperature . Note that  is also an output of COSMOTherm calculations. As mentioned before, this methodology serves as a good approximation since the results in many cases have limited precision, especially for acentric factors.

## Supporting Information Available

Group contribution parameters for all the group contribution models introduced in the present article (XLSX)
Experimental solvation Gibbs energy data used to parametrize the PR/COSMO-RS EoS and their estimation from this EoS (XLSX)
List of Functional Groups: UNIFAC functional groups (Table S1), specific groups for free radicals (Table S2), specific groups for transition states (Table S3) (PDF)
