# Supplementary material for: Integrating Solvent Effects into the Prediction of Kinetic Constants Using a COSMO-Based Equation of State
Source: J Chem Theory Comput. 2025 Mar 25;21(7):3625–48. doi: 10.1021/acs.jctc.5c00133 (PMC11984481; doi:10.1021/acs.jctc.5c00133)
Supplement: Supplementary file 3 — ct5c00133_si_003.pdf [file ct5c00133_si_003.pdf]

Integrating Solvent Effects into the Prediction of Kinetic Constants Using a COSMO-based Equation-of-State

Francisco Paes[1], Gabriel de Souza Batalha[1], Fabiola Citrangolo Destro[1], René Fournet[1], Romain Privat[1(*)], Jean-Noël Jaubert[1(*)], Baptiste Sirjean[1(*)]

[1] Université de Lorraine, CNRS, LRGP, F-54000 Nancy, France

SUPPORTING INFORMATION. LIST OF FUNCTIONAL GROUPS

The following tables present the list of UNIFAC groups, along with their respective versions for functional groups with radical sites and the groups developed for transition states. The numerical values of the parameters for each group are given in the other supporting information files (in XLSX format).

Table S1. *List of UNIFAC functional groups.*

#	Group	#	Group	#	Group	#	Group	#	Group
1	[CH_3]	31	[$CHNH_2$]	61	[CH_2SH]	91	[AMH_2]	121	[Cl_2]
2	[CH_2]	32	[CH_3NH]	62	[FURFURAL]	92	[$AMHCH_3$]	122	[NH_3]
3	[CH]	33	[CH_2NH]	63	[DOH]	93	[$AMHCH_2$]	123	[SO_2]
4	[C]	34	[CHNH]	64	[I]	94	[$AM(CH3)_2$]	124	[C(=O)OOH]
5	[CH2=CH]	35	[CH_3N]	65	[BR]	95	[$AMCH_3CH_2$]	125	[CH_2OOH]
6	[CH=CH]	36	[CH_2N]	66	[CH≡C]	96	[$AM(CH_2)_2$]	126	[C-O-OH]
7	[CH_2=C]	37	[$ACNH_2$]	67	[C≡C]	97	[$C_2H_5O_2$]	127	[CHOOH]
8	[CH=C]	38	[C_5H_5N]	68	[DMSO]	98	[$C_2H_4O_2$]	128	[$CHOOCH_2$]
9	[C=C]	39	[C_5H_4N]	69	[ACRY]	99	[CH_3S]	129	[CH_3OOC]
10	[ACH]	40	[C_5H_3N]	70	[CL-(C=C)]	100	[CH_2S]	130	[CH_2ONO_2]
11	[AC]	41	[CH_3CN]	71	[ACF]	101	[CHS]	131	[$CHONO_2$]
12	[$ACCH_3$]	42	[CH_2CN]	72	[DMF]	102	[MORPH]	132	[H_2C=O]
13	[$ACCH_2$]	43	[COOH]	73	[CF_3]	103	[C_4H_4S]	133	[CH_3OOH]
14	[ACCH]	44	[HCOOH]	74	[CF_2]	104	[C_4H_3S]	134	[CH_2CCO]
15	[OH]	45	[CH_2CL]	75	[CF]	105	[C_4H_2S]	135	[H_2O_2]
16	[CH3OH]	46	[CHCL]	76	[COO]	106	[H_2]	136	[C2H5OH]
17	[H_2O]	47	[CCL]	77	[SIH_3]	107	[O_2]	137	[CH3C=OCH3]
18	[ACOH]	48	[CH_2CL_2]	78	[SIH_2]	108	[N_2]	138	[CH3COOH]
19	[CH_3CO]	49	[$CHCL_2$]	79	[SIH]	109	[CO]	139	[CH3CH=O]
20	[CH_2CO]	50	[CCL_2]	80	[SI]	110	[CO_2]		
21	[CH=O]	51	[$CHCL_3$]	81	[SIO]	111	[H_2S]		
22	[CH_3COO]	52	[CCL_3]	82	[NMP]	112	[CH_4]		
23	[CH_2COO]	53	[CCL_4]	83	[CCL_3F]	113	[C_2H_2]		
24	[HCOO]	54	[ACCL]	84	[CCL2F]	114	[C_2H_4]		
25	[CH_3O]	55	[CH_3NO_2]	85	[$HCCL_2F$]	115	[C_2H_6]		
26	[CH_2O]	56	[CH_2NO_2]	86	[HCCLF]	116	[C_3H_6]		
27	[CHO]	57	[$CHNO_2$]	87	[$CCLF_2$]	117	[C_3H_8]		
28	[THF]	58	[$ACNO_2$]	88	[$HCCLF_2$]	118	[C_4H_{10}]		
29	[CH_3NH_2]	59	[CS_2]	89	[$CCLF_3$]	119	[C_3H_4]		
30	[CH_2NH_2]	60	[CH_3SH]	90	[CCL_2F_2]	120	[Ar]		

Table S2. *List of specific groups for free radicals.*

#	Correction group	#	Correction group
H-atom		*Phenyl*	
1	$[H^{\bullet}]$	23	$[AC^{\bullet}]$
Acetylenic		*Primary*	
2	$[C^{\bullet} \equiv C]$	24	$[C^{\bullet}H_2]$
3	$[H_2C^{\bullet}]$	25	$[C_4^{\bullet}H_9]$butane-primary
Alkoxyl		26	$[C_2^{\bullet}H_5]$ethane-primary
4	$[O^{\bullet}]$	27	$[C^{\bullet}H_3]$ methane-primary
5	$[HO^{\bullet}]$	28	$[C^{\bullet}H_3OH]$ methanol-primary
6	$[CH_3O^{\bullet}]$ methanol	29	$[C_3^{\bullet}H_7]$propane-primary
7	$[C_2H_5O^{\bullet}]$ ethanol	30	$[C_3^{\bullet}H_5]$propene-primary
8	$[ACO^{\bullet}]$		
9	$[ArO^{\bullet}]$phenol	*Secondary*	
Benzyl		31	$[C^{\bullet}H]$
10	$[ACC^{\bullet}H_2]$	32	$[C_4^{\bullet}H_9$butane-secondary
11	$[ACC^{\bullet}H]$	33	$[C^{\bullet}H]$resonance
Carbonyl and carboxyl		34	$[C_3^{\bullet}H_7]$propane-secondary
12	$[C^{\bullet} = O]$	*Tertiary*	
13	$[COO^{\bullet}]$	35	$[C^{\bullet}]$
14	$[C^{\bullet}OO]$	*Vinyl*	
15	$[HC^{\bullet} = O]$	36	$[C^{\bullet} = C]$
16	$[CH_3C^{\bullet} = O]$	37	$[CH^{\bullet} = C]$
17	$[CH_3COO^{\bullet}]$	38	$[CH^{\bullet} = CH_2]$ ethene-vinyl
18	$[HCOO^{\bullet}]$ formicacid	39	$[CH^{\bullet}CC = O]$ ethenone-vinyl
19	$[C^{\bullet}OOH]$ formicacid	40	$[C^{\bullet} = CH]$
Peroxyl		41	$[CH_2 = C^{\bullet}]$
20	$[HO_2^{\bullet}]$ hydrogen-peroxide	42	$[C_3^{\bullet}H_5]$propene-vinyl
21	$[C(=O)OO^{\bullet}]$		
22	$[CHOO^{\bullet}]$		
23	$[CH_3OO^{\bullet}]$ methylhydroperoxide		
24	$[C_2H_5OO^{\bullet}]$ ethylhydroperoxide		
21	$[CH_2OO^{\bullet}]$		
22	$[C - O - O^{\bullet}]$		

Table S3. *List of specific groups for transition states.*

#	RO—H—R type	#	ROO—H—R type
1	$[CH_3O\ -H-CH_2]$	1	$[CH_2OO\ -H-CH_2]$
2	$[CH_2O\ -H-CH_2]$	2	$[CHOO\ -H-CH_2]$
3	$[CHO\ -H-CH_2]$	3	$[COO\ -H-CH_2]$
4	$[CO-H-CH_2]$	4	$[CH_3OO\ -H-C]$
5	$[CH_3O\ -H-CH]$	5	$[CH_2OO\ -H-C]$
6	$[CH_2O\ -H-CH]$	6	$[CHOO\ -H-C]$
7	$[CHO-H-CH]$	7	$[COO\ -H-C]$
8	$[CO\ -H-CH]$	8	$[CH_3OO\ -H-CH]$
		9	$[CH_2OO\ -H-CH]$
		10	$[CHOO\ -H-CH]$
		11	$[COO\ -H-CH]$
